# Correction: Role of L-Carnitine supplementation on rate of weight gain and biomarkers of Environmental Enteric Dysfunction in children with severe acute malnutrition: A protocol for a double-blinded randomized controlled trial

**DOI:** 10.1371/journal.pone.0307982

**Published:** 2024-07-24

**Authors:** Jinat Alam, Md. Ridwan Islam, Shah Mohammad Fahim, Md. Amran Gazi, Tahmeed Ahmed

In the Sample size and power subsection of Materials and methods, there is an error in the equation in the third sentence. Please view the complete, correct equation here:

$$n=2\times {\left(\frac{Z1‐\alpha +Z1‐\beta }{\delta ‐\delta }\right)}^{2}\times S^{2}$$
