# Supplementary material for: Role of L-Carnitine supplementation on rate of weight gain and biomarkers of Environmental Enteric Dysfunction in children with severe acute malnutrition: A protocol for a double-blinded randomized controlled trial
Source: PLoS One. 2022 Sep 30;17(9):e0275291. doi: 10.1371/journal.pone.0275291 (PMC9524645; doi:10.1371/journal.pone.0275291)
Supplement: S1 File — (DOC) [file pone.0275291.s001.doc]

29 July 2021

**Ethical Review Committee**

# Approval Letter

To: Dr Jinat Alam

Principal Investigator of research protocol # PR-21046 Nutrition and Clinical Services Division (NCSD)

From: Professor Ahmed Abu Saleh Chairperson

Ethical Review Committee (ERC)

Sub: Approval of research protocol # PR-21046 Approval Date: 29 July 2021

Expiration Date: 28 July 2022

Review Type: Full Committee Review Risk Level: No more than minimal Project type: New Project

Thank you for your memo dated 29 July 2021 attaching the modified version of your research protocol # PR-21046 entitled “Role of L-Carnitine supplementation on rate of weight gain and biomarkers of Environmental Enteric Dysfunction (EED) in children with severe acute malnutrition”; version No. 1.0; version date 24 May 2021; addressing the issues raised by the ERC in its 23rd Virtual ERC meeting held on 06 July 2021 to the satisfaction of the Committee. I am pleased to inform you that your protocol is approved. You will be required to observe the following terms and conditions in implementing the research protocol:

1. The research protocol is approved for 12-month period from the date of approval of the protocol by the Ethical Review Committee. The Federal regulations require review of an approved study not less than once per 12- month period. To comply with federal regulations, a continuing review application must be submitted to the IRB Secretariat for this study to continue beyond 28 July 2022.

All necessary materials for continuing review must be reviewed with sufficient time for review and issuing continued approval before the expiration date. Failure to initiate a continuing review application in a timely fashion may result in discontinuation of study activities until approval can be renewed. Performing study activities, including data analysis, beyond the expiration date results in noncompliance of federal regulations.

1. The ERC approval shall automatically be revoked after one year if the protocol is not started. After one year, you shall have to seek approval for revalidation of the protocol by the ERC before starting.
2. You should notify the IRB Secretariat of the start date of the protocol for updating in the integrated Navision system. The protocol start date will not be updated in the Navision system until receiving information from you. Therefore, you will not be able to operate budget code and continue spending funds under the research protocol.
3. As Principal Investigator, the ultimate responsibility for scientific and ethical conduct including the protection of the rights and welfare of study participants vest upon you. You shall also be responsible for ensuring competence, integrity and ethical conduct of other investigators and staff directly involved in this research protocol.
4. You shall conduct the study in accordance with the ERC-approved protocol and shall fully comply with any subsequent determinations by the ERC.
5. You shall obtain prior approval from the Research Review Committee and the ERC for any modification in the approved research protocol and/or approved consent form(s), except in case of emergency to safeguard/ eliminate apparent immediate hazards to study participants. Such changes must immediately be reported to the ERC Chairman.
6. You shall recruit/enrol participants for this study strictly adhering to the criteria mentioned in the research protocol.
7. You shall obtain legally effective informed consent (i.e. consent should be free from coercion or undue influence) from the selected study participants or their legally responsible representative, as approved in the protocol, using the approved consent form prior to their enrolment in this study. Before obtaining consent, all prospective study participants must be adequately informed about the purpose(s) of the study, its methods and procedures, and also what would be done if they agree and also if they do not agree to participate in the study.
8. They must be informed that their participation in the study is voluntary and that they can withdraw their participation any time without any prejudice. Signed consent forms should be preserved for a period of at least five years following official termination of the study.
9. You shall promptly report the occurrence of any Serious Adverse Event or unanticipated problems of potential risk to study participants or others to the ERC in writing within 24 hours of such occurrences.
10. Any significant new findings, developing during the course of this study that might affect the risks and benefits and thus influence either participation in the study or continuation of participation should be reported in writing to the participants and the ERC.
11. You shall report progress of research to the ERC for continuing review of the implementation of the research protocol as stipulated in the ERC Guidelines. Relevant excerpt of ERC Guidelines and ‘*Annual/Completion* Report for Research Protocol involving Human Subjects’ are attached for your information and guidance.

***Page 2 of 3***

1. Data and/or samples should be collected and interviews should be conducted, as specified in the ERC-approved protocol, and confidentiality must be maintained. Data/samples must be protected by reasonable security, safeguarding against risks such as their loss or unauthorized access, destructions, used by others, and modification or disclosure of data. Data/samples should not be disclosed, made available to or use for purposes other than those specified in the protocol, and shall be preserved for a period, as specified under Centre's policies/practices.
2. You shall promptly and fully comply with the decision of the ERC to suspend or withdraw its approval for the research protocol.
3. The ERC should be immediately notified if the protocol is discontinued before the expected date of completion.

**Approved documents**:

- 1. Protocol version No 1.0 dated 24 May 2021
  2. English and Bangla Information Sheet for Parent/Legal Guardian of the Participant Main Trial; version No 1.0 dated 24 May 2021
  3. English and Bangla Consent Form for Parent/Legal Guardian of the Participant Main Trial; version No 1.0 dated 24 May 2021
  4. English and Bangla Role of L-Carnitine supplementation on rate of weight gain and biomarkers of Environmental; version No 1.0 dated 24 May 2021
  5. English and Bangla Enteric Dysfunction (EED) in children with severe acute malnutrition; version No 1.0 dated 24 May 2021

The IRB of icddr,b shall take into account the regulations of the Bangladesh Medical Research Council (BMRC), WHO, international guidelines for biomedical research as laid down by the Council of International Organization of Medical Sciences (CIOMS), the Declaration of Helsinki in relation to biomedical research involving human participants, ICH Guidelines on Good Clinical Practice (GCP), National Institutes of Health (NIH), National Institute of Allergy and Infectious Diseases (NIAID), and Division of Microbiology and Infectious Diseases (DMID). If there is any new declaration involving human participants, contents of such declaration should be appropriately adhered to and the applicable laws and policies of the local government.

I wish you success in running the above-mentioned study. Cc: Senior Director, NCSD

Senior Manager, Budget & Planning, Finance

# Memorandum

29 July 2021

To: Professor Ahmed Abu Saleh

Chairperson

Ethical Review Committee (ERC)

From: Dr Jinat Alam

Principal Investigator of research protocol # PR-21046

Nutrition and Clinical Services Division (NCSD)

Sub: Responding to the comments from ERC for the approval of Research protocol # PR-21046

Thank you respected reviewers for your valuable comments on our research protocol # PR-21046 titled “Role of L-Carnitine supplementation on rate of weight gain and biomarkers of Environmental Enteric Dysfunction (EED) in children with severe acute malnutrition”. We have addressed the raised issues on the protocol and revised the protocol as suggested.

1. RRC approval “yes/no” and date are missing.

Response: Thank you sir for your valuable observation and review. We have filled up the section of our protocol as suggested (Please see page-01).

1. Signatures of the PI, Co-PI, Co-I and Division Director are missing where applicable in the RRC face sheet.

Response: Thank you sir for your valuable review. We have revised our protocol with the signature of the Principal Investigators (Please see page-08, 09 & 70). Co-PI, Co-I, and Division director’s approval and written consent were taken through email due to COVID-19 lockdown situation (Please see page-71 & 72).

1. The proposal does not include any capacity build up, therefore this statement in RRC face sheet should be taken out. PI or investigator’s development will not be applicable here.

Response: Thank you very much sir for your valuable advice. We have included the capacity build up section in the protocol (Please see page-01).

1. Consent forms:
2. The title of the information sheet / consent form is incorrect. It should be information sheet / consent form for parent / legal guardian.

Response: Thank you very much sir for your valuable suggestions. We did correction to our information sheet and consent form as suggested (Please see page-48 & 51 ).

1. English and Bangla text under section “why invited to participate” needs revision. Investigators started the section as “To fulfil the aim of our research study, we need to collect blood, urine, and stool sample from your child” which is scaring for parent / guardian. It should go in the procedure. They should start the section like “we are inviting you for participation of your child in our study as he / she is suffering from SAM and aged 9-24 months. You are looking for children like him/her for our study”.

Response: Thank you very much sir for your valuable suggestions and opinion. We did correction to our consent form (Both English and Bangla) as suggested (Please see page-48 & 53).

1. The text in the bullets of the informed consent sounds like parent / guardian is the participant of the study. Therefore, the texts there need revision.

Response: Thank you so much sir for your valuable observation and review. We revised our consent form (Both English and Bangla) as suggested (Please see page-51 & 55).

Thank you very much.

Cc: Senior Director, NCSD.

# Ethical Review Committee Comments Letter

Memorandum

12 July 2021

To: Dr Jinat Alam

Principal Investigator of research protocol # PR-21046

Nutrition and Clinical Services Division (NCSD)

From: Professor Ahmed Abu Saleh

Chairperson

Ethical Review Committee (ERC)

Sub: Research protocol # PR-21046

Thank you very much for submitting your research protocol # 21046, titled “Role of L-Carnitine supplementation on rate of weight gain and biomarkers of Environmental Enteric Dysfunction (EED) in children with severe acute malnutrition” for consideration in its 23rd Virtual ERC meeting held on 06 July 2021. After review and discussion, the Committee made following observations on it:

a) RRC approval “yes/no” and date are missing.

b) Signatures of the PI, Co-PI, Co-I and Division Director are missing where applicable in the RRC face sheet.

c) The proposal does not include any capacity build up, therefore this statement in RRC face sheet should be taken out. PI or investigator’s development will not be applicable here.

d) Consent forms:

1. The title of the information sheet / consent form is incorrect. It should be information sheet / consent form for parent / legal guardian.
2. The English and Bangla text under section “why invited to participate” needs revision. Investigators started the section as “To fulfil the aim of our research study, we need to collect blood, urine, and stool sample from your child” which is scaring for parent / guardian. It should go in the procedure. They should start the section like “we are inviting you for participation of your child in our study as he / she is suffering from SAM and aged 9-24 months. You are looking for children like him/her for our study”.
3. The text in the bullets of the informed consent sounds like parent / guardian is the participant of the study. Therefore, the texts there need revision.

Please modify the protocol addressing the above observations and submit a revised version of the protocol for consideration of the Chair.

Thank you.

Cc: Senior Director, NCSD

*Page 2 of 2*

**Memorandum**

Date: 02 July, 2021

To: Chairperson

Ethical Review Committee (ERC)

icddr,b

Through: Dr Tahmeed Ahmed

Executive Director and Senior Director

Nutrition and Clinical Services Division (NCSD)

icddr,b

From: Dr Jinat Alam,

Project Research Physician

Nutrition and Clinical Services Division (NCSD) and

Principal Investigator of the research protocol #PR-21046

Subject: Submission of new research protocol # PR-21046 for ERC review

We would like to submit the attached research protocol titled **“**Role of L-Carnitine supplementation on rate of weight gain and biomarkers of Environmental Enteric Dysfunction (EED) in children with severe acute malnutrition (Protocol # PR-21046, Version No-1.00, Version Date- 24.05.2021)” to the Ethical Review Committee. Through this study we would like to assess the role of L-Carnitine supplementation on malnourished children. This study will be a double-blinded, placebo-controlled, randomized clinical trial. Our study site will be Nutritional Rehabilitation Unit (NRU) of Dhaka Hospital of icddr,b.

We have received approval from the Research Review Committee on 01 July, 2021. We are therefore requesting you to consider the protocol for review and approval of the Ethical Review Committee. Thank you very much for your consideration.

Enclosed documents:

1. RRC Approval Letter
2. RRC comments and Response
3. Protocol
4. Approved budget from finance
5. Biography of the investigators
6. Consent forms in English and Bengali
7. External Reviewer’s comments and response
8. Gender analysis tool
9. Questionnaire in English
10. Email approval from Co-Investigator

**ERC Face Sheet**

| Principal Investigator: Dr. Jinat Alam | | | Date: 02.07.2021 |
| --- | --- | --- | --- |
| Protocol Number: | | P | R | - | 2 | 1 | 0 | 4 | 6 | | --- | --- | --- | --- | --- | --- | --- | --- | | Version No. 1.00; Version date: 24.05.2021 | |
| Protocol Title: Role of L-Carnitine supplementation on rate of weight gain and biomarkers of Environmental Enteric Dysfunction (EED) in children with severe acute malnutrition | | | |

**Check the appropriate box to answer to each of the following**

| 1. | Type of Protocol: | | | | | | | | | | 6. | Participants will be informed about: | | | | Yes | No | NA |
| --- | --- | --- | --- | --- | --- | --- | --- | --- | --- | --- | --- | --- | --- | --- | --- | --- | --- | --- |
|  | New Study  Secondary data analysis (Skip 3, 5 & 6)  Umbrella Project (If yes, submit an overview)  Student protocol | | | | | | | | | |  | (a) | | Nature and purposes of the study | |  |  |  |
|  |  |  |  |  |  |  |  |  |  |  |  | (b) | | Procedures to be followed including  available alternatives | |  |  |  |
|  |  |  |  |  |  |  |  |  |  |  |  | (c) | | Risk- physical, social, psychological | |  |  |  |
|  | | | | | | | | | | |  | (d) | | Sensitive questions | |  |  |  |
| 2. | | Study population: | | | Yes | | | | No | |  | (e) | | Benefits to be derived | |  |  |  |
|  | | (a) | | Ill participants |  | | | |  | |  | (f) | | Right to refuse to participate or to withdraw from the study | |  |  |  |
|  |  | (b) | | Non-ill participants |  | | | |  | |  |  |  |  |  |  |  |  |
|  |  | (c) | | Minor or persons under guardianship |  | | | |  | |  | (g) | | Confidential handling of data | |  |  |  |
|  |  | d) | | Others _______________________ |  | | | |  | |  | (h) | | Provision for compensation | |  |  |  |
|  |  |  |  |  |  |  |  |  |  |  |  |  |  |  |  |  |  |  |
| 3. | | Does the study involve: | | | Yes | | | | No | |  | | | | | | | |
|  | | (a) | | Physical risk to participants | |  | | |  | | 7. | | Precautions to be taken to protect anonymity of study participants | | | Yes | No | NA |
|  |  | (b) | | Social risk to participants | |  | | |  | |  |  |  |  |  |  |  |  |
|  |  | (c) | | Psychological risks to participants | |  | | |  | |  | | | | |  |  |  |
|  |  | (d) | | Discomfort to participants | |  | | |  | | 8. | | **The following have been included** | | | Yes | No | NA |
|  |  | (e) | | Invasion of participants’ privacy | |  | | |  | |  | | (a) | | ERC Summary |  |  |  |
|  |  | (f) | | Disclosure of information damaging to participants or others | |  | | |  | |  |  | (b) | | Consent form for adult participants |  |  |  |
|  |  |  |  |  |  |  |  |  |  |  |  |  | (c) | | Consent form from parent or guardian |  |  |  |
|  | | | | | | | | | | |  |  | (d) | | Assent form |  |  |  |
| 4. | | | Does the study involve use of : | | | Yes | | | No | |  |  | (e) | | Consent^a^ form of previous studies |  |  |  |
|  |  |  |  |  |  |  |  |  |  |  |  |  | (f) | | MOU^b^ |  |  |  |
|  | | | (a) | Body fluids or organs | |  | | |  | |  |  | (g) | | MTA^b^ |  |  |  |
|  |  |  | (b) | Fetal tissue or abortus | |  | | |  | |  |  | (g) | | Questionnaire/Research instrument^c^ |  |  |  |
|  |  |  | (c) | Records (hospital, medical, death or other) | |  | | |  | |  | | | | | | | |
|  |  |  | (d) | Stored biological specimens | |  | | |  | |  |  |  |  |  |  |  |  |
|  |  |  | (e) | Data from Previous study | |  | | |  | |  |  |  |  |  |  |  |  |
|  | | | | | | | | | | |  | | ^a^ If data from previous studies will be used  ^b^ The document must be submitted to the ERC for approval before study commencement  ^c^ If the final version is not ready, a draft version should be submitted. The final version must be approved by the ERC before data collection | | | | | |
| 5 | | | Informed written consent/assent be obtained from: | | | | Yes | No | | NA |  |  |  |  |  |  |  |  |
|  | | | (a) | Adult participants | | |  |  | |  |  |  |  |  |  |  |  |  |
|  |  |  | (b) | Parent or guardian or next to kin (if  participants are <11 years of age /or  under guardianship) | | |  |  | |  |  |  |  |  |  |  |  |  |
|  |  |  | (c) | Participants aged 11 – 17 years (Assent) | | |  |  | |  |  |  |  |  |  |  |  |  |

I agree to abide by the approved protocol and shall obtain prior approval of the ERC for any changes in the protocol.

Principal Investigator

#### ERC Abstract Summary

Title: Role of L-Carnitine supplementation on rate of weight gain and biomarkers of Environmental Enteric Dysfunction (EED) in children with severe acute malnutrition

Principal Investigator: Dr Jinat Alam

1. **Purpose and procedures:**

Globally an estimated 14.3 million under-5 children are severely malnourished, which is an underlying cause of nearly half of global deaths in under-5 children despite standardized rehabilitation protocols. It is associated with high relapse rates following discharge. Moreover, malnourished children suffer from deficiencies of several essential nutrients. Carnitine is one of the essential nutrients and plays a critical role in β-oxidation of fatty acids and energy production. It is important for improvement of heart and brain function, muscle movement, and many other body processes as well as disease prevention. The consequences of nutritional impairment can be perilous if carnitine deficiency is coupled with Environmental Enteric Dysfunction (EED). EED is found to be associated with childhood growth faltering and impaired cognitive development. Recent evidence postulated an association between EED and secondary carnitine deficiency in malnourished children. Carnitine deficiency leading to EED may negatively affect the growth and development of young children. However, evidence on carnitine status and its consequences in relation to EED in diarrheal children with SAM is very limited in Bangladesh. There may have specific biological obstacles in relation to EED which render nutritional therapy ineffective or partially effective in malnourished children. Therefore, this study is proposed to assess the carnitine level and explore its role on rate of weight gain, duration of hospital stays and EED biomarkers in children with SAM. This study will be a double-blinded, placebo-controlled randomized clinical trial.

1. **Requirement for a specific population**:

The study population will be consist of children, aged 9-24 months, who presented with an acute episode of diarrhea and severe acute malnutrition (SAM). Based on statistical considerations, the study aims to recruit 98 participants, including 49 children in intervention arm and 49 children in placebo arm.

During literature review we found, malnourished children had lower serum carnitine, particularly among those with severe acute malnutrition as well as those children suffer from Environmental enteric dysfunction. Moreover, both EED and SAM are pervasively common in Bangladeshi children. Several years ago few studies was done at this age group and found significant rate of weight gain and improvement of their conditions. They proved the rationale of L-carnitine syrup as a treatment of malnutrition at this age group.

Children will be excluded if they have severe sepsis or septic shock, already taking medications containing L-carnitine, have Tuberculosis, congenital defects or chromosomal anomalies, or a diagnosed case of Thalassemia or have an active or previous history of convulsion.

1. **Consent/Assent process**:

Informed written consent will be obtained from parents or caregiver at NRU (Nutritional Rehabilitation Unit) and the consent process will explain total background of the study, procedures, risks, benefits, compensations and right to withdraw or not to enroll in the study.

1. **Potential risks**:

There is no major risk involved in participation of the enrolled children in this study. Possible adverse events may be nausea, vomiting, diarrhea. Despite taking precautions, if the enrolled child develops any symptoms due to this study procedure, we would provide appropriate treatment at the Dhaka Hospital of icddr,b. The child may experience little discomfort or pain while providing the blood samples. There is no major risk involved in giving blood samples.

1. **Potential benefits**:

The enrolled children will be directly and indirectly benefited from participating in the study. We are assuming due to L-carnitine deficiency, enrolled child is suffering from SAM and EED. Through this study, the parents of the study participants will be able to know the current status of L-carnitine and EED of their child. Moreover, the children would be able to contribute to our understanding to develop more effective treatments for malnutrition and new treatment for EED. In the long term, the results of this study would benefit other children in Bangladesh and elsewhere by helping us understand the effects of L-carnitine syrup supplementation in malnutrition and EED. The goal is to identify the role of L-carnitine syrup on the rate of weight gain and biomarkers of EED in children with SAM.

1. **Confidentiality**:

We do hereby state that privacy, anonymity and confidentiality of data/information identifying the children will strictly be maintained. We would keep all medical information, description of treatment, and results of the laboratory tests performed in the enrolled children confidential, under lock and key, and none other than our research staff will have an access to this information. No one other than this group of investigators, regulatory authorities and the Ethical Review Committee (a group of experts which protects the interest of study participants) of icddr,b and investigators sponsor of this study would have access to such information. The child’s name and identity will not be disclosed while analyzing or publishing the results of this study.

1. **Privacy**:

In case of use of the information collected from this study, privacy, anonymity and confidentiality of information will be strictly maintained.

1. **Time required:**

Children will be enrolled at this study when transferred to NRU for rehabilitation after completion of acute phase management. At NRU, trained research staff will observe each child for 60 minutes following ingestion of investigational products and record adverse event, if any. Children will also get standard treatment of malnutrition as per icddr,b management protocol. We will give supplementation for total 15 days. If the study participants fulfil the discharge criteria before completion of 15 days supplementation we will discharge the participants with remaining doses for total 15 days and advised them to come for follow up after completion of remaining doses.

1. **Compensation**:

The children enrolled in the study will receive free treatment like every patient of the hospital. Similarly, we will not pay money to the study participants for participation in our study. If any child has a study related injury s/he will receive standard care at the Dhaka Hospital (Cholera Hospital) in Mohakhali, Dhaka. If any participants needs to come for follow up in to our hospital we will only provide them the travel allowances.

1. **Interview/Questionnaire**:

The study involves interview at the time of enrolment at NRU. Questionnaires will contain information about medical history including nature and duration of illness, medication for current illness; socio-demographic characteristics such as- age, sex, religion, parental age with education, parents occupation, monthly family income, number of siblings etc. Information will also be collected about child’s feeding practice such as- history of breast feeding, formula or other complementary feeding, immunization status and recent or past history of respiratory infections. No sensitive questions will be asked.

1. **Use of records/data**:

We will use the data collected from the enrolled patients upon ensuring confidentiality, privacy and anonymity.

1. **Use of specimens**:

We will collect biological sample (stool, urine and 5.0 ml of blood) on the enrolment day as well as after completion of 15 days of supplementation which will help us to assess carnitine level of the child and also assess the effect of L-carnitine supplementation on rate of weight gain and also on biomarkers of EED. We will store the sample for future studies at icddr,b laboratory for 5 years under the ownership of icddr,b.

1. **Use of photographs, video and audio records**:

We would not be collecting video or audio records in our study.

1. **Counseling:**

Counselling session for the mother or caregiver will be arranged in the NRU to help understanding the benefit of this study.

1. **Patient management or referral:**

All the study participants will be managed as per routine management guidelines of icddr,b. If referral is required, it will be done as per hospital guidelines.

**Attachment C of Annex-I**

**Memorandum**

01 July 2021

To: Dr Jinat Alam

Principal Investigator of research protocol # PR-21046 Nutrition and Clinical Services Division (NCSD)

From: Shafiqul Alam Sarker, MD, Ph.D, FRCP Chairperson

Research Review Committee (RRC)

Sub: Approval of research protocol # PR-21046

Thank you for your memo dated 30 June 2021 attaching the modified version of your research protocol # PR-21046 titled “Role of L-Carnitine supplementation on rate of weight gain and biomarkers of Environmental Enteric Dysfunction (EED) in children with severe acute malnutrition”; version no. 1.0, version date 24 May 2021; addressing the issues raised by the committee in its 20th Virtual RRC meeting held on 03 June 2021 to the satisfaction of the Committee. Accordingly, the Committee approved the research protocol to proceed subject to the approval of the Ethical Review Committee (ERC).

**Terms of approval**

1. The research protocol is approved for 12-month period from the date of approval of the protocol by the Ethical Review Committee. Approval for further continuation of the research work, if needed, shall be obtained before expiration of the initial approval.
2. You should notify the IRB Secretariat of the start date of the protocol for updating in the integrated navision system. The protocol start date will not be updated in the navision system until receiving information from you. Therefore you will not be able to operate budget code and continue spending funds under the research protocol.
3. The RRC approval shall automatically be revoked after one year if the protocol is not started. After one year, you shall have to seek approval for revalidation of the protocol by the RRC & ERC before starting the protocol.
4. This approval is only valid whilst you hold a position at icddr,b; and in the event of your departure from the Centre, a new Principal Investigator will be designated for the research protocol. ***Page 1 of 2***
5. You should notify the RRC and the ERC immediately of any serious or unexpected adverse effects on participants or unforeseen events that might affect continued acceptability of the protocol.
6. Any changes to the research protocol require the submission (in prescribed form) and approval of an amendment/addendum. Substantial variations may require a new protocol.
7. Continued approval of this protocol is dependent on your periodically updating the Centre’s database for the protocol to show the progress; and a final report/completion report should be submitted at the conclusion of the protocol.
8. You shall submit a report for time extension of the protocol (in prescribed form) if you are unable to complete the protocol activities within the time mentioned in the protocol.
9. You are responsible for systematic storage and retention of the original data pertaining to the research protocol; and the ownership of data after certain period shall be determined as per Centre’s rules and regulations.
10. The RRC should be notified if the protocol is discontinued before the expected date of completion.

I wish you all the success in conducting the research protocol. Thank you.

Cc: Senior Director, NCSD

Senior Manager, Budget & Planning, Finance

***Page 2 of 2***

Memorandum

30 June 2021

To: Shafiqul Alam Sarker, MD, PhD, FRCP

Chairperson

Research Review Committee (RRC)

From: Dr Jinat Alam

Principal Investigator of Research Protocol # PR-21046

Nutrition and Clinical Services Division (NCSD)

Sub: Responding to the comments from RRC for the approval of Research Protocol#PR-21046

Thank you respected reviewers for your valuable comments on our research protocol # PR-21046 titled “Role of L-Carnitine supplementation on rate of weight gain and biomarkers of Environmental Enteric Dysfunction (EED) in children with severe acute malnutrition”. We have addressed the raised issues on the protocol and revised the protocol as suggested.

1. The impact of L-carnitine supplementation on biomarkers of EED among malnourished children is not known, albeit EED can be ameliorated by L- carnitine supplementation as it is linked to carnitine deficiency. This study to assess carnitine level and explore its role on the rate of weight gain (anthropometric measurements), duration of hospital stays, and biomarkers of EED in severely malnourished children in Bangladesh.

Response: Thank you sir for your valuable observations and considerations.

1. The protocol has been reviewed by two external reviewers and they have commented on the protocol nicely and the investigators have addressed those comments. This study will be a double-blinded, placebo-controlled randomized clinical trial where the children in intervention arm will receive L- carnitine supplementation for 15 days. The control arm will receive placebo for the same duration. This study will be conducted only in SAM children admitted to Dhaka Hospital, icddr,b .

Response: Thank you sir for your valuable observations and considerations.

1. MAM is also a problem in Bangladesh and there is no good guideline for the treatment of MAM in the Community. It would have been nice if the investigators could have another one arm of this study where they could enrol MAM children. These children could be given the same intervention and could be followed up in the same way.

Response: Thank you sir for your valuable suggestion. This is an excellent point. Yes, we completely agree that as there is no established guideline for the treatment of MAM, including a MAM arm would certainly be beneficial and interesting. But due to our budget constraint we couldn’t include MAM in our study design. Nevertheless, we hope to explore this topic of research interest in the future.

1. Children will receive 15 days L-carnitine supplementation. It is not mentioned in the protocol from where the L-carnitine will be procured for this study. Little more detail of the investigational product needs to be given in the protocol. This is not clear because child will be provided L-carnitine supplementation in the NRU for 15 days and child will stay entire period at NRU. How do you estimate the role of L-carnitine supplementation on duration of hospital stay?

Response: Thank you sir for your valuable comment. We have communicated with a pharmaceutical company in Bangladesh regarding procurement of both the L-carnitine syrup and placebo. They have assured us verbally that they will be able to provide us with enough syrup and placebo to complete the study efficiently. After the official confirmation we will include all the details in our protocol.

According to our study design, after completing the acute phase treatment, our study participants will shift to NRU in order to start their rehabilitation phase treatment. They will receive their first dose of supplementation on Day 1 of NRU stay after enrolment. During this intervention period if study participants fulfill the discharge criteria of NRU (WLZ/WHZ ≥ -2SD, WAZ ≥ -3SD and oedema resolved), we will discharge the patient with remaining dose of the syrup and advise them to come for follow up after completion of the remaining dose. The same method will be applicable for the placebo arm. By this way, we will estimate the role of L-carnitine supplementation on duration of hospital stay. We have included all the details in our protocol as suggested (Please see Page-21).

1. Sample size calculation is based on the primary outcome weight gain but, they have not mentioned any unit of weight gain there. Just mentioned that “mean difference between the rate of weight gain after supplementation was 2.4”. They should mention here the unit and when they will see this difference.

Response: Thank you sir for your valuable advice. The unit of weight gain from baseline was in kg. We have mentioned it in our protocol as advised (Please see page-19). We will measure the difference after completion of 15 days of supplementation of investigational product.

1. A lot of laboratory tests will be required for this study including the L-Carinitine in plasma and urine. They have not mentioned in the protocol in which lab these tests will be carried out.

Response: Thank you sir for your valuable comment. We will do all the laboratory investigations (LC-MS and ELISA) at icddr,b laboratory and one of our co-investigators Md Amran gazi will supervise these tests. Remaining two tests (S. creatinine and Potential Renal Solute Load) will be done at icddr,b diagnostic laboratory. We have mentioned the name of laboratory in our protocol as suggested. (Please see page-19)

1. In page 5, under consent process: The consenting process would be only written, not both written and oral.

Response: Thank you very much sir for your suggestion. We have corrected the point as advised in page-5.

1. In page 6, under biological specimens: In response to the query - ‘What types of tests will be carried out with the preserved specimens?’ the answers were ‘For Future Microbiome Studies’ which is not correct. Should be revisited.

Response: Thank you sir for your comment. As suggested the previous answer has been corrected in page-6.

1. In page 57 and 66: Appendix number and protocol number is missing.

Response: Thank you sir for your valuable comment. We have included the appendix number and protocol number in page 57 and 65 as advised.

1. What is the mode of action of the L-carnitine in malnutrition? The L-carnitine is burning the fat and usually the malnourished children have low fat. How the researchers expecting impact of L-carnitine in malnourished children? If the L-carnitine help in increasing the weight gain, then the mechanism should be explicitly described in the protocol.

Response: Thank you sir for your valuable opinion. The mode of action of the L-carnitine is shown in the protocol by a figure- The Carnitine Shuttle (Figure-1) (Please see page-14). The Carnitine Shuttle shows, carnitine helps in transfer of Acyl Co-A into mitochondrial matrix from cytosol. By this way, carnitine helps in energy production in the form of ATP through β-oxidation pathway.

Several studies found that ATP has beneficial effects on nutritional status. Therefore, it is assumed that carnitine may help in increasing weight gain by increasing the ATP production. Few studies also suggest, there is a positive correlation present between albumin and plasma carnitine levels in protein energy malnutrition. An incremental growth was observed in 22 out of 33 carnitine-administered patients who presented with failure to thrive this was attributed to the role of carnitine as a muscle growth factor. Thus L-carnitine supplementation may help in increasing weight gain in SAM children. We have mentioned this mechanism in the protocol (Please see page 14-15).

1. In the inclusion criteria, the investigators mentioned that they will include “diarrheal children with SAM aged 9-24 months”, how long will be patients be kept in hospital to get weight gain? Should be explicitly described in the methods section.

Response: Thank you sir for your valuable comment. The patient will be discharged as per the discharged criteria of NRU (WLZ/WHZ ≥ -2SD, WAZ ≥ -3SD and oedema resolved). We have mentioned it in our methods section as suggested (Please see page-21).

1. In some of the cases there might be genetically metabolic disorder, how will those patients be identified and excluded from the study?

Response: Thank you sir for your valuable opinion. Usually, a genetically metabolic disorder is diagnosed by using screening test- from blood test or examination of a tissue sample to determine whether a specific enzyme is deficient or missing. Sometimes after birth many of these disorders are also detected by routine new-born screening tests. Genetic testing is also used. Due to budget constraint, it wouldn’t be possible for us to do such expensive test. We will try to exclude the possible cases by taking history (Presence of any family history of these conditions, as these conditions can be passed down from parents or having any report of new-born screening test, if done before). We will also do physical examinations (unusual facial appearance, eye disease, and unusual enlargement of liver or spleen, and repeated hypoglycaemia) and will try to exclude the participants with possibility of having genetically metabolic disorders.

1. Many technical terms have been used in the information sheet for patients. The language of this ‘Information Sheet’ should be made simple to make it easily understandable for the study participants.

Response: Thank you sir for your valuable suggestion. We have revised the “Information Sheet” as suggested, so that it would be easily understandable to study participants (Please see page-48).

1. A big questionnaire/study tools have been attached with the protocol. Many of the questionnaire are redundant and not related with the study objectives. The irrelevant questions should be deleted from the questionnaire.

Response: Thank you sir for your valuable suggestion. We have revised our questionnaires form as suggested (Please see page-57).

Thank you very much.

Cc: Senior Director, NCSD

**Memorandum**

18 June 2021

To: Dr Jinat Alam

Principal Investigator of research protocol # PR-21046 Nutrition and Clinical Services Division (NCSD)

From: Shafiqul Alam Sarker, MD, PhD, FRCP Chairperson

Research Review Committee (RRC)

Sub: Research protocol # PR-21046

Thank you for submitting your research protocol # PR-21046 titled “Role of L- Carnitine supplementation on rate of weight gain and biomarkers of Environmental Enteric Dysfunction (EED) in children with severe acute malnutrition” for consideration of the RRC and present it before the Committee in its 20th Virtual RRC meeting held on 03 June 2021. This is to inform you that after review and discussion, the committee made the following observations on the protocol:

1. The impact of L-carnitine supplementation on biomarkers of EED among malnourished children is not known, *albeit* EED can be ameliorated by L- carnitine supplementation as it is linked to carnitine deficiency. This study to assess carnitine level and explore its role on the rate of weight gain (anthropometric measurements), duration of hospital stays, and biomarkers of EED in severely malnourished children in Bangladesh
2. The protocol has been reviewed by two external reviewers and they have commented on the protocol nicely and the investigators have addressed those comments. This study will be a double-blinded, placebo-controlled randomized clinical trial where the children in intervention arm will receive L- carnitine supplementation for 15 days. The control arm will receive placebo for the same duration. This study will be conducted only in SAM children admitted to Dhaka Hospital, icddr,b .
3. MAM is also a problem in Bangladesh and there is no good guideline for the treatment of MAM in the Community. It would have been nice if the investigators could have another one arm of this study where they could enroll MAM children. These children could be given the same intervention and could be followed up in the same way.
4. Children will receive 15 days L-carnitine supplementation. It is not mentioned in the protocol from where the L-Carnitine will be procured for this study. Little more detail of the investigational product needs to be given in the protocol. This is not clear because child will be provided L-carnitine supplementation in the NRU for 15 days and child will stay entire period at NRU. How do you estimate the role of L-carnitine supplementation on duration of hospital stay?
5. Sample size calculation is based on the primary outcome weight gain but, they have not mentioned any unit of weight gain there. Just mentioned that “mean difference between the rate of weight gain after supplementation was 2.4”. They should mention here the unit and when they will see this difference.
6. A lot of laboratory tests will be required for this study including the L- Carinitine in plasma and urine. They have not mentioned in the protocol in which lab these tests will be carried out.
7. In page 5, under consent process: The consenting process would be only written, not both written and oral.
8. In page 6, under biological specimens: In response to the query - ‘What types of tests will be carried out with the preserved specimens?’ the answers were ‘For Future Microbiome Studies’ which is not correct. Should be revisited.
9. In page 57 and 66: Appendix number and protocol number is missing.
10. What is the mode of action of the L-carnitine in malnutrition? The L-carnitine is burning the fat and usually the malnourished children have low fat. How the researchers expecting impact of L-carnitine in malnourished children? If the L-carnitine help in increasing the weight gain, then the mechanism should be explicitly described in the protocol.
11. In the inclusion criteria, the investigators mentioned that they will include “diarrheal children with SAM aged 9-24 months”, how long will be patients be kept in hospital to get weight gain? Should be explicitly described in the methods section.
12. In some of the cases there might be genetically metabolic disorder, how will those patients be identified and excluded from the study?
13. Many technical terms have been used in the information sheet for patients. The language of this ‘Information Sheet’ should be made simple to make it easily understandable for the study participants.
14. A big questionnaire/study tools have been attached with the protocol. Many of the questionnaire are redundant and not related with the study objectives. The irrelevant questions should be deleted from the questionnaire.

***Page 2 of 3***

You are, therefore, advised to address each of the above-mentioned observations of the committee and submit the revised version of the protocol for consideration by the chair.

Thank you once again.

CC: Senior Director, NCSD

**Memorandum**

Date: May 24, 2021

To: Dr Shafiqul Alam Sarker, MD, PhD, FRCP

Chairperson

Research Review Committee (RRC)

icddr,b

Through: Dr Tahmeed Ahmed

Executive Director and Senior Director

Nutrition and Clinical Services Division (NCSD)

icddr,b

From: Dr Jinat Alam,

Project Research Physician

Nutrition and Clinical Services Division (NCSD) and

Principal Investigator of the research protocol #PR-21046

Subject: Submission of new research protocol # PR-21046 for RRC review

We would like to submit the attached research protocol titled **“**Role of L-Carnitine supplementation on rate of weight gain and biomarkers of Environmental Enteric Dysfunction (EED) in children with severe acute malnutrition (Protocol # PR-21046)” to the Research Review Committee. Through this study we would like to assess the role of L-Carnitine supplementation on malnourished children. This study will be a double-blinded, placebo-controlled, randomized clinical trial. Our study site will be Nutritional Rehabilitation Unit (NRU) of Dhaka Hospital of icddr,b.

We have received comments from the external reviewers and responded to their comments. We are therefore requesting you to consider the protocol for review and approval of the Research Review Committee. Thank you very much for your consideration.

Enclosed documents:

1. Protocol
2. Approved budget from finance
3. Biography of the investigators
4. Consent forms in English and Bengali
5. External Reviewer’s comments and response
6. Gender analysis tool
7. Questionnaire in English
8. Email approval from Co-Investigators

|  | | | | | RRC APPLICATION FORM | | | | |
| --- | --- | --- | --- | --- | --- | --- | --- | --- | --- |
| RESEARCH PROTOCOL **Number: PR - 21046**  **Version No. 1.00**  **Version date: 24-05-2021** | **FOR OFFICE USE ONLY** | | | | | | | | |
|  | RRC Approval: | | | | Yes | | No | | Date: 01-07-2021 |
|  | ERC Approval: | | | | Yes | | No | | Date: 29-07-2021 |
|  | AEEC Approval: | | | | Yes | | No | | Date: |
|  | External IRB Approval | | | | Yes | | No | | Date: |
|  | Name of External IRB: ___________________________________________ | | | | | | | | |
| **Protocol Title:* (**maximum 250 characters including space**)**  Role of L-Carnitine supplementation on rate of weight gain and biomarkers of Environmental Enteric Dysfunction (EED) in children with severe acute malnutrition | | | | | | | | | |
| **Short Title: (**maximum **100** characters including space)  L-Carnitine supplementation, rate of weight gain and EED in children with SAM | | | | | | | | | |
| **Key Words:** Malnutrition; L-Carnitine; Environmental Enteric Dysfunction; Under-5 children; Bangladesh | | | | | | | | | |
| **Name of the Research Division Hosting the Protocol:***  Health Systems and Population Studies Division (HSPSD)  Nutrition and Clinical Services Division (NCSD)  Infectious Diseases Division (IDD) | | | Maternal and Child Health Division (MCHD)  Laboratory Sciences and Services Division (LSSD)  Other (specify) ____________________________ | | | | | | |
| **Has the Protocol been Derived from an Activity:***  No  Yes (please provide following information):  Activity No.:  Activity Title:  PI:  Grant No.:       Budget Code:       Start Date:       End Date: | | | | | | | | | |
| **icddr,b Strategic Priority/ Initiative (SP 2015-8):* (**check all that apply**)** | | | | | | | | | |
| Reducing maternal and neonatal mortality  Controlling enteric and respiratory infections  Preventing and treating maternal and childhood malnutrition  Detecting and controlling emerging and re-emerging infections | | | Achieving universal health coverage  Examining the health consequences of climate change  Preventing and treating non-communicable diseases  Others (specify) ________________________________ | | | | | | |
| **Research Phase (4 Ds):* (**check all that apply**)**  Discovery  Development | | | Delivery  Evaluation of Delivery | | | | | | |
| **Anticipated Impact of Research:* (**check all that apply and please provide details below**)**  Knowledge Production  Capacity Building | | | Informing Policy  Health and Health Sector Benefits  Economic Benefits | | | | | | |
| **Please provide details here:**  Results of this study would generate evidence regarding L-carnitine supplementation in the management of severe acute malnutrition and EED  This study will augment the research capacity of junior researchers and clinicians involved in the study. Moreover, we will perform all the laboratory analysis in our laboratory at icddr,b. We will conduct Enzyme-linked immunosorbent assay (ELISA) to determine carnitine level in blood and urine samples collected from the study participants. This will help to develop in-house capacity to perform ELISA and train our laboratory personnel accordingly using the platform of this study. Moreover, the results of this study would generate knowledge in management of malnourished children with carnitine deficiency and EED. Thus it would enhance the capacity of healthcare professionals in low-inclome settings to diagnose and prevent the adverse consequences in relation to carnitine deficiency and EED in children with SAM. | | | | | | | | | |
| **Which of the Sustainable Development Goal This Protocol Relates to?:* (**check all that apply**)**  1. End poverty in all its forms everywhere  2. End hunger, achieve food security and improved nutrition and promote sustainable agriculture  3. Ensure healthy lives and promote well-being for all at all ages  4. Ensure inclusive and equitable quality education and promote lifelong learning opportunities for all  5. Achieve gender equality and empower all women and girls  6. Ensure availability and sustainable management of water and sanitation for all  7. Ensure access to affordable, reliable, sustainable and modern energy for all  8. Promote sustained, inclusive and sustainable economic growth, full and productive employment and decent work for all  9. Build resilient infrastructure, promote inclusive and sustainable industrialization and foster innovation  10. Reduce inequality within and among countries  11. Make cities and human settlements inclusive, safe, resilient and sustainable  12. Ensure sustainable consumption and production patterns  13. Take urgent action to combat climate change and its impacts  14. Conserve and sustainably use the oceans, seas and marine resources for sustainable development  15. Protect, restore and promote sustainable use of terrestrial ecosystems, sustainably manage forests, combat desertification, and halt and reverse land degradation and halt biodiversity loss  16. Promote peaceful and inclusive societies for sustainable development, provide access to justice for all and build effective, accountable and inclusive institutions at all levels  17. Strengthen the means of implementation and revitalize the global partnership for sustainable development | | | | | | | | | |
| **Does this Protocol Use the Gender Framework:***  (Please visit: <http://shetu.icddrb.org/index.php?option=com_content&view=article&id=265&Itemid=677> for Gender Alanysis Tool with instructions) | | | Yes (please complete Gender Analysis Tool)  No | | | | | | |
| If ‘no’ is the response, its reason(s) in brief: | | | | | | | | | |
| **Will this Research Specifically Benefit the Disadvantaged (**economically, socially and/or otherwise**):** | | | | | | | | Yes  No | |
| **Does this Protocol use Behaviour Change Communication:** | | | | | | | | Yes  No | |
| **Principal Investigator (Should be icddr,b staff):*** Sex Female  Male  Dr Jinat Alam  Project Research Physician, Maternal and Child Nutrition, NCSD, icddr,b  Cell: +88 01676742255  Email: [jinat.alam@icddr,b.org](mailto:jinat.alam@icddr,b.org)  Do you have ethics certification?  No  Yes (please attach in your CV below)  Do you have RBM training certification?  No  Yes (please attach the certificate with CV below) | | | | | | Primary Scientific Division of the PI Nutrition and Clinical Services Division | | | |
| **Co-Principal Investigator(s) Internal**: Sex Female  Male  Dr Shah Mohammad Fahim  Research Investigator  Maternal and Child Nutrition, NCSD, icddr,b  Phone: +880-2-9827001-10, Ext: 2287  Cell: +88 01912682540  Email: [mohammad.fahim@icddr,b.org](mailto:mohammad.fahim@icddr,b.org)  Signature or written consent of Co-PI: __________________  (electronic signature or email or any sort of written consent)  [if more than one, please copy and paste this row for additional Co-PIs]  Do you have ethics certification?  No  Yes (please attach in your CV below)  Do you have RBM training certification?  No Yes (please attach the certificate with CV below) | | | | | | Primary Scientific Division/ Programme of the Co-PI Nutrition and Clinical Services Division  ___________________________  Approval of the Respective Senior Director/ Programme Head  (Signature) | | | |
| **Co-Principal Investigator(s) - External:** Sex Female  Male    Address (provide full official address, including land phone no(s), extension no. (if any), cell phone number, and email address).    Signature or written consent of Co-PI: __________________  (electronic signature or email or any sort of written consent)  [if more than one, please copy and paste this row for additional Co-PIs] | | | | | | | | | |
| **Co-Investigator(s) Internal**: Sex Female  Male  Dr Tahmeed Ahmed  Executive Director, icddr,b  Phone: +880-2-9827001-10 Ext: 2300  Cell: +88 01713044799  Email: [tahmeed@icddr,b.org](mailto:tahmeed@icddr,b.org)  Signature or written consent of Co-I: __________________  (electronic signature or email or any sort of written consent)  [if more than one, please copy and paste this row for additional Co-Is]  Do you have ethics certification?  No  Yes (please attach in your CV below)  Do you have RBM training certification?  No  Yes (please attach the certificate with CV below) | | | | | | Primary Scientific Division/ Programme of the Co-I Nutrition and Clinical Services Division  ___________________________  Approval of the Respective Senior Director/ Programme Head  (Signature) | | | |
| **Co-Investigator(s) - Internal:** Sex  Female  Male  Md Amran Gazi  Assistant Scientist  Maternal and Child Nutrition, NCSD, icddr,b  Cell: +88 01680731163  Email: [amran.gazi@icddr,b.org](mailto:amran.gazi@icddr,b.org)  Signature or written consent of Co-I: __________________  (electronic signature or email or any sort of written consent)  [if more than one, please copy and paste this row for additional Co-Is  Do you have ethics certification?  No  Yes (please attach in your CV below)  Do you have RBM training certification?  No  Yes (please attach the certificate with CV below) | | | | | | Primary Scientific Division of the Co-I Nutrition and Clinical Services Division  ___________________________  Approval of the Respective Senior Director/ Programme Head  (Signature) | | | |
| **Co-Investigator(s) - Internal:** Sex  Female  Male  Dr Md Ridwan Islam  Study Physician  Maternal and Child Nutrition, NCSD, icddr,b  Cell: +88 01718531403  Email: [ridwan.islam@icddr,b.org](mailto:ridwan.islam@icddr,b.org)  Signature or written consent of Co-I: __________________  (electronic signature or email or any sort of written consent)  [if more than one, please copy and paste this row for additional Co-Is  Do you have ethics certification?  No  Yes (please attach in your CV below)  Do you have RBM training certification?  No  Yes (please attach the certificate with CV below) | | | | | | Primary Scientific Division of the Co-I Nutrition and Clinical Services Division  ___________________________  Approval of the Respective Senior Director/ Programme Head  (Signature) | | | |
| **Co-Investigator(s) – External:** Sex  Female  Male  **Address** (provide full official address, including land phone no(s), extension no. (if any), cell phone number, and email address):    Signature or written consent of Co-I: __________________  (electronic signature or email or any sort of written consent)  [if more than one, please copy and paste this row for additional Co-Is] | | | | | | | | | |
| **Student Investigator(s) - Internal:**  Sex  Female  Male    (Position, phone no, extension no, cell, and email address **):**    Signature or written consent of Student Investitor: __________________  (electronic signature or email or any sort of written consent)  Have ethics certificate?  No  Yes (If Yes, please attach to your CV below) | | | | | | Students Affiliation  ___________________________  Approval of the Respective Senior Director/ Programme Head    (Signature) | | | |
| **Student Investigator(s) - External:** Sex  Female  Male  Address (provide full official address, including land phone no(s), extension no. (if any), cell phone number, and email address):    Signature or written consent of Student Investitor: __________________  (electronic signature or email or any sort of written consent) | | | | | | | | | |
| **Student Investigator(s) - External:** Sex  Female  Male  Address (provide full official address, including land phone no(s), extension no. (if any), cell phone number, and email address):    Signature or written consent of Student Investitor: __________________  (electronic signature or email or any sort of written consent) | | | | | | | | | |
| **Collaborating Institute(s):** Please provide full official address   | Country |  | | --- | --- | | Contact person |  | | Department  (including Division, Centre, Unit) |  | | Institution  (with official address) |  | | Directorate  (in case of GoB i.e. DGHS) |  | | Ministry (in case of GoB) |  |   **Institution # 1** | | | | | | | | | |
| **Institution # 2**   | Country |  | | --- | --- | | Contact person |  | | Department  (including Division, Centre, Unit) |  | | Institution  (with official address) |  | | Directorate  (in case of GoB | i.e. DGHS) | | Ministry (in case of GoB) |  | | | | | | | | | | |
| **Institution # 3**   | Country |  | | --- | --- | | Contact person |  | | Department  (including Division, Centre, Unit) |  | | Institution  (with official address) |  | | Directorate  (in case of GoB i.e. DGHS) |  | | Ministry (in case of GoB) |  |   Note: If less than or more than three collaborating institutions, please delete or insert blocks as needed. | | | | | | | | | |
| **Contribution by the Members of the Scientific Team:**   | Members’ Name | Contribution | | | | | | | | | | --- | --- | --- | --- | --- | --- | --- | --- | --- | --- | | Research idea/  concept | Study design | Protocol writing | Respond to external reviewers’ comments | Defending at IRB | Developing data collection Tool(s) | Data Collection | Data analysis/ interpretation of results | Manuscript writing | | Dr Jinat Alam |  |  |  |  |  |  |  |  |  | | Dr Shah Mohammad Fahim |  |  |  |  |  |  |  |  |  | | Dr Tahmeed Ahmed |  |  |  |  |  |  |  |  |  | | Md Amran Gazi |  |  |  |  |  |  |  |  |  | | Dr Md Ridwan Islam |  |  |  |  |  |  |  |  |  | |  |  |  |  |  |  |  |  |  |  | |  |  |  |  |  |  |  |  |  |  | |  |  |  |  |  |  |  |  |  |  | |  |  |  |  |  |  |  |  |  |  | |  |  |  |  |  |  |  |  |  |  | | | | | | | | | | |
| Study Population: Sex, Age, Special Group and Ethnicity   | **Research Subject:**  Human  Animal  Microorganism  Other (specify): _____________  **Sex:**  Male  Female  Transgender  **Age:**  0 – 4 years  5 – 10 years  11 – 17 years  18 – 64 years  65 + | **Special Group:**  Pregnant Women  Fetuses  Prisoners  Destitutes  Service Providers  Cognitively Impaired  CSW  Expatriates  Immigrants  Refugee  Others (specify): ________________  **Ethnicity:**  No ethnic selection (Bangladeshi)  Bangalee  Tribal group  Other (specify): ________________ | | --- | --- |   **NOTE:** It is icddr.b’s policy to include men, women, children and transgender in its research projects involving participation of humans, unless there is strong justification(s) for their exclusion. | | | | | | | | | |
| **Consent Process: (**Check all that apply**)**  Written  Oral  Audio  Video  None | | **Language:**  Bangla  English  Other (specify: _______________ | | | | | | | |
| **Project/Study Site: (**Check all that apply**)**  Chakaria  Bandarban  Dhaka Hospital  Kamalapur Field Site/HDSS  Mirpur (Dhaka)  Matlab DSS Area  Matlab non-DSS Area  Matlab Hospital  Mirzapur | | Bianibazar (Sylhet)  Kanaighat (Sylhet)  Jakigonj (Sylhet)  Other community in Dhaka  Name: _________________________________  Other sites in Bangladesh  Name: _________________________________  Multi-national Study  Name of the country______________________ | | | | | | | |
| **Project/Study Type: (Check all that apply)**   | Case Control Study  Clinical Trial (Hospital)*  Community-based Trial/Intervention  Cross Sectional Survey  Family Follow-up Study  Longitudinal Study (cohort or follow-up)  Meta-analysis  Programme Evaluation | Programme (Umbrella Project)  Prophylactic Trial  Record Review  Secondary Data Analysis  Protocol No. of Data Source: _____________  Surveillance/Monitoring  Systematic Review  Other (specify): _________________________ | | --- | --- |   ***Note**: International Committee of Medical Journal Editors (ICMJE) defines Clinical Trial as “*Any research project that prospectively assigns human participants to intervention and comparison groups to study the cause-and-effect relationship between a medical intervention and a health outcome*”.  PI of the RRC- and ERC-approved Clinical Trials should provide necessary information to IRB Secretariat (Research Administration) for registration and uploading into relevant websites (usually at the <https://register.clinicaltrials.gov/>). They should also provide relevant information to the IRB Secretariat in the event of amendment/modification after their approval by RRC and ERC.  In case of a multi-country study and if a study is registered elsewhere by the prime recipient or others; it does not need to be re-registered under icddr,b’s account; provided evidence of NCT registration number is submitted to the IRB. | | | | | | | | | |
| **Biological Specimen:** | |  | | | | | | | |
| 1. Will the biological specimen be stored for future use? | | | | Yes  No  Not applicable | | | | | |
| 1. If the response is ‘yes’, how long the specimens will be preserved? | | | | 5 years | | | | | |
| 1. What types of tests will be carried out with the preserved specimens? | | | | Enzyme-linked immunosorbent assay (ELISA) | | | | | |
| 1. Will the consent be obtained from the study participants for use of the preserved specimen for other initiative(s) unrelated to this study, without their re-consent? | | | | Yes  No  Not applicable | | | | | |
| 1. Will the specimens be shipped to other country/ countries?   If yes, name of institution(s) and country/countries. | | | | Yes  No  Not applicable  Name ____________________________________ | | | | | |
| 1. If shipped to another country, will the surplus/unused specimen be returned to icddr,b?   If the response is ‘no’, then the surplus/unused specimen must be destroyed. | | | | Yes  No  Not applicable | | | | | |
| 1. Who will be the custodian of the specimen at icddr,b? | | | | Dr Jinat Alam | | | | | |
| 1. Who will be the custodian of the specimen when shipped outside Bangladesh? | | | | Not applicable | | | | | |
| 1. Who will be the owner(s) of the specimens? | | | | icddr,b | | | | | |
| 1. Has a MoU been signed with regards to collection, storage, use and ownership of specimen?   If the response is ‘yes’, please attach a copy of the MoU.  If the response is ‘no’, appropriate justification should be provided for not signing a MoU. | | | | Yes  No  Not applicable | | | | | |
| **Proposed Sample Size:**  Sub-group (Name of subgroup e.g. Men, Women) and Number   | Name | Number | | --- | --- | | (1) Group A (Intervention arm) | 49 | | (2) Group B (Control arm) | 49 | | **Total sample size** | 98 | | | | | | | | | | |
| **Determination of Risk: Does the Research Involve (Check all that apply**)   | Human exposure to radioactive agents?  Foetal tissue or abortus?  Investigational new device?  Specify: __________________  Existing data available from Co-investigator? | Human exposure to infectious agents?  Investigational new drug?  Existing data available via public archives/sources?  Pathological or diagnostic clinical specimen only?  Observation of public behaviour?  New treatment regime? | | --- | --- | | | | | | | | | | |
| | Will the information be recorded in such a manner that study participants can be identified from the information directly or through identifiers linked to the study participants? | Yes | No | | --- | --- | --- | | Does the research deal with sensitive aspects of the study participants’ sexual behaviour, alcohol use or illegal conduct such as drug use? | Yes | No |   **Could information on study participants, if available to people outside of the research team:**   | 1. Place them at risk of criminal or civil liability? | Yes | No | | --- | --- | --- | | 1. Damage their financial standing, reputation or employability, or social rejection, or lead to stigma, divorce etc.? | Yes | No | | | | | | | | | | |
| **Do you consider this research:** (check one)   | Greater than minimal risk | No more than minimal risk | Only part of the diagnostic test | | --- | --- | --- |   **Note: Minimal Risk:** The probability and the magnitude of the anticipated harm or discomfort to participants is not greater than those ordinarily encountered in daily life or during the performance of routine physical, psychological examinations or tests, e.g. the risk of drawing a small amount of blood from a healthy individual for research purposes is no greater than when the same is performed for routine management of patients. | | | | | | | | | |
| **Risk Group of Infectious Agent and Use of Recombinant DNA** | | | |  | | | | | |
| 1. Will specimens containing infectious agent be collected? | | | | Yes  No  Not applicable | | | | | |
| 1. Will the study involve amplification by culture of infectious agents? | | | | Yes  No  Not applicable | | | | | |
| 1. If response to questions (a) and/or (b) is ‘yes’, to which Risk Group (RG) does the agent(s) belong? (Please visit <http://shetu.icddrb.org/index.php?option=com_content&view=article&id=265&Itemid=677> to review list of microorganism by Risk Group) | | | | RG1  RG2  RG3  RG4 | | | | | |
| 1. Does the study involve experiments with recombinant DNA? | | | | Yes  No  Not applicable | | | | | |
| **Does the study involve any biohazards materials/agents or microorganisms of risk group 2, 3, or 4 (GR2, GR-3 or GR4)?**  Yes  No  [If the response is ‘yes’] I, (print name of the PI) affirm that we will use the standard icddr,b laboratory procedures for biosafety of the hazardous materials/agents or microorganisms in the conduction of the study.  24.05.2021  **Signature of the Principal Investigator Date** | | | | | | | | | |
| **Dissemination Plan:** [please explicitly describe the plans for dissemination, including how the research findings would be shared with stakeholders, identifying them if known, and the mechanism to be used; anticipated type of publication (working papers, internal (institutional) publication, international publications, international conferences/seminars/workshops/ agencies. [Check all that are applicable]   | **Dissemination type** | **Response** | | **Description (if the response is a yes)** | | --- | --- | --- | --- | | Seminar for icddr,b scientists/ staff | No | Yes | We will disseminate research findings in the Executive Director’s seminar for icddr,b scientists/staff | | Internal publication | No | Yes |  | | Working paper | No | Yes |  | | Sharing with GoB (e.g. DGHS/ Ministry, others) | No | Yes | The final results, when published, will be shared with the government officials | | Sharing with national NGOs | No | Yes |  | | Presentation at national workshop/ seminar | No | Yes |  | | Presentation at international workshop/ conference | No | Yes | The results will be presented in an international conference | | Peer-reviewed publication | No | Yes | The findings will be published in an international peer-reviewed journal | | Sharing with international agencies | No | Yes |  | | Sharing with donors | No | Yes | Results will be shared with the donor | | Policy brief | No | Yes |  | | Other |  |  |  | | Other |  |  |  | | | | | | | | | | |
| `  Funding:   | Is the protocol fully funded? | Yes | No | | --- | --- | --- | | If the answer is yes, please provide sponsor(s)’s name | 1. | | | 2. | | | Is the protocol partially funded? | Yes | No | | If the answer is yes, please provide sponsor(s)’s name | 1. | | | 2. | |   If fund has not been identified:   | Is the proposal being submitted for funding? | Yes | No | | --- | --- | --- | | If yes, name of the funding agency | 1. Nestle Foundation | | |  | | | | | | | | | | | |
| | **Conflict of interest:**  Do any of the participating investigators and/or member(s) of their immediate families have an equity relationship (e.g. stockholder) with the sponsor of the project or manufacturer and/or owner of the test product or device to be studied or serve as a consultant to any of the above? | | | --- | --- | | No | Yes (please submit a written statement of disclosure to the Executive Director, icddr,b) |   **Proposed Budget:**  Dates of Proposed Period of Support Cost Required for the Budget Period ($)  (Day, Month, Year - DD/MM/YY)   | **Years** | **Direct Cost** | **Indirect Cost** | **Total Cost** | | --- | --- | --- | --- | | **Year-1** | 9,342 | 0 | 9,342 | | **Year-2** | 15,658 | 0 | 15,658 | | **Year-3** | - | - | - | | **Year-4** | - | - | - | | **Year-5** | - | - | - | | **Total** | 25,000 | 0 | 25,000 |     Beginning Date : 19.10.2021    End Date : 30.09.2022 | | | | | | | | | |
| **Certification by the Principal Investigator:**  I certify that the statements herein are true, complete and accurate to the best of my knowledge. I am aware that any false, fictitious, or fraudulent statements or claims may subject me to criminal, civil, or administrative penalties. I agree to accept the responsibility for the scientific conduct of the project and to provide the required progress reports including updating protocol information in the NAVISION if a grant is awarded as a result of this application.  I also certify that I have read icddr,b Data Policies and understand the PIs’ responsibilities related to archival and sharing of research data, and will remain fully compliant to the Policies. (Note: The Data Policies can be found here: <http://shetu.icddrb.org/index.php?option=com_content&view=article&id=273&Itemid=685>)  24.05.2021  **Signature of PI Date** | | | | | | | | | |
| **Approval of the Project by the Division Director of the Applicant:**  The above-mentioned project has been discussed and reviewed at the Division level.   | Dr Tahmeed Ahmed | ___________ |  | | --- | --- | --- | | Name of the Division Director | Signature | Date of Approval | | | | | | | | | | |

List of abbreviations

A1AT: Alpha-1 anti-trypsin

AGP: Alpha-1-acid glycoprotein

ATP: Adenosine triphosphate

BDHS: Bangladesh Demographic and Health Survey

CRF: Case Record Form

CRP: C-reactive protein

DSMP: Data Safety Monitoring Plan

EED: Environmental Enteric Dysfunction

ERC: Ethical Review Committee

GCP: Good Clinical Practice

icddr,b: International Centre for Diarrheal Disease Research, Bangladesh

ICU: Intensive Care Unit

ICH: International Council of Harmonization

KT ratio: Kynurenine: tryptophan ratio,

LSU: Longer Stay Unit

MPO: Myeloperoxidase

MUAC: Mid-upper arm circumference

NCSD: Nutrition and Clinical Services Division

NEO: Neopterin

NRU: Nutritional Rehabilitation Unit

PRSL: Potential Renal Solute Load

RRC: Research Review Committee

SAM: Severe Acute Malnutrition

S. Cr: Serum creatinine

SOPs: Standard Operating Procedures

WAZ: Weight-for-Age z-score

WHO: World Health Organization

WHZ: Weight-for-Height z-score

WLZ: Weight-for-Length z-score

WMA: The World Medical Association

Table of Contents

[RRC APPLICATION FORM 1](#__RefHeading___Toc336858679)

[Project Summary 12](#__RefHeading___Toc336858680)

[Hypothesis to be tested:](#__RefHeading___Toc336858681) 13

[Specific Objectives: 1](#__RefHeading___Toc336858682)3

[Background of the Project including Preliminary Observations:](#__RefHeading___Toc336858683) 13

[Research Design and Methods](#__RefHeading___Toc336858684) 15

[Sample Size Calculation and Outcome (Primary and Secondary) Variable(s) 1](#__RefHeading___Toc336858685)9

[Data Analysis 2](#__RefHeading___Toc336858686)2

[Data Safety Monitoring Plan (DSMP)](#__RefHeading___Toc336858687) 22

[Ethical Assurance for Protection of Human rights 2](#__RefHeading___Toc336858688)3

[Use of Animals 2](#__RefHeading___Toc336858689)4

[Collaborative Arrangements 2](#__RefHeading___Toc336858690)4

[Facilities Available 2](#__RefHeading___Toc336858691)4

[Literature Cited 2](#__RefHeading___Toc336858692)4

[Budget](#__RefHeading___Toc336858693) 26

[Other Support](#__RefHeading___Toc336858694) 28

[Biography of the Investigators](#__RefHeading___Toc336858695) 36

[Format for Consent Form](#__RefHeading___Toc336858696) 43

[Check-List](#__RefHeading___Toc336858697) 57

Check here if appendix is included

| Appendix 1 | External reviewer’s comments and response | Page: 29 |
| --- | --- | --- |
| Appendix 2 | Gender analysis tools | Page: 35 |
| Appendix 3 | Questionnaires | Page: 43 |
| Appendix 4 | WMA Declaration of Helsinki - Ethical Principles for Medical Research Involving Human Subjects | Page: 52 |

| Project Summary [The summary, within a word limit of 300, should be stand alone and be fully understandable.] |
| --- |
| **Principal Investigator:** Dr Jinat Alam |
| **Research Protocol Title:** Role of L-Carnitine supplementation on rate of weight gain and biomarkers of Environmental Enteric Dysfunction (EED) in children with severe acute malnutrition |
| Proposed start date: 01/09/2021 Estimated end date: 31/08/2023 |
| **Background (brief):**   1. **Burden:**   Globally, an estimated 14.3 million under-5 children are severely malnourished. Two-thirds of them live in Asian countries including Bangladesh. Acute malnutrition is an underlying cause of nearly half of global deaths in under-5 children despite standardized rehabilitation protocols. It is also associated with high relapse rates following discharge.   1. **Knowledge Gap:**   Malnourished children suffer from deficiencies of several essential nutrients. Studies showed that malnourished children had lower serum carnitine level and demonstrated its role on the rate of weight gain in children with severe acute malnutrition (SAM). The consequences of nutritional impairment can be perilous if carnitine deficiency is coupled with Environmental Enteric Dysfunction (EED). Recent evidence confirms that EED is characterized by secondary carnitine deficiency in children. Carnitine deficiency leading to EED may cause childhood growth faltering and impaired cognitive development. However, evidence on carnitine status and its consequences in relation to EED in diarrheal children with SAM is very limited in Bangladesh.   1. **Relevance:**   Such lack of information regarding the role of L-carnitine in improving the rate of weight gain in malnourished children susceptible to EED is an obstacle in limiting the relapse and adverse consequences of SAM in diarrheal children living in resource limited countries.  **Hypothesis:** L- carnitine supplementation for 15 days in children with SAM will improve the rate of weight gain and biomarkers of EED  **Objective:** To investigate the role of L-carnitine supplementation on the rate of weight gain, duration of hospital stays and EED biomarkers among children with severe acute malnutrition  **Methods:** This study will be a double-blinded, placebo-controlled randomized clinical trial where the children in intervention arm will receive L-carnitine supplementation for 15 days. The control arm will receive placebo for the same duration. We will enroll diarrheal children aged 9-24 months suffering from SAM both in intervention and control groups. This study will be conducted at Dhaka Hospital of icddr,b.  **Outcome measures/variables:**   - Primary outcome variable: Rate of weight gain - Secondary outcome variables:   1. Duration of hospital stays  2. EED biomarkers [myeloperoxidase (MPO), neopterin (NEO), alpha-1 anti-trypsin (A1AT), kynurenine: tryptophan (KT) ratio, and citrulline] |

**Description of the Research Project**

## Hypothesis to be tested:

| In a hypothesis testing research proposal, briefly mention the hypothesis to be tested and provide the scientific basis of the hypothesis, critically examining the observations leading to the formulation of the hypothesis. |
| --- |

Does this research proposal involve testing of hypothesis:  No Yes (describe below)

L-carnitine supplementation for 15 days in children with severe acute malnutrition will improve the rate of weight gain and biomarkers of EED

## Specific Objectives:

| Describe the specific objectives of the proposed study. State the specific parameters, gender aspects, biological functions, rates, and processes that will be assessed by specific methods. |
| --- |

## To investigate the role of L-carnitine supplementation on the rate of weight gain among the children with SAM

## To investigate the role of L-carnitine supplementation on the duration of the hospital stays

## To examine the role of L-carnitine supplementation on EED biomarkers, for instance, myeloperoxidase (MPO), neopterin (NEO), alpha-1 anti-trypsin (A1AT), kynurenine: tryptophan (KT) ratio, and citrulline in children with SAM

## Background of the Project including Preliminary Observations:

| Provide scientific validity of the hypothesis based on background information of the proposed study and discuss previous works on the research topic, including information on sex, gender and diversity (ethnicity, SES) by citing specific references. Critically analyze available knowledge and discuss the questions and gaps in the knowledge that need to be filled to achieve the proposed aims. If there is no sufficient information on the subject, indicate the need to develop new knowledge. |
| --- |

Carnitine is a low molecular weight naturally occurring compound, found in all mammalian species (1, 2). L-carnitine (levocarnitine; 3-hydroxy-4-N-trimethylammonium butyrate) is a standard biologically active form of carnitine (1, 2). It is one of the essential nutrients that plays a critical role in the β-oxidation of fatty acids and energy production in the form of adenosine triphosphate (ATP) (3-5). Carnitine is important for the improvement of heart and brain function, muscle movement, and many other body processes as well as disease prevention (3, 4). It is synthesized in the liver, kidney, and brain from essential amino acids lysine and methionine (4, 6). It is found mainly in animal-source foods, such as red meat, chicken, fish, and dairy products, whereas only a negligible amount is available in plant source foods (4). In normal individuals, the homeostasis is achieved and maintained by a combination of oral absorption from dietary sources, *de novo* biosynthesis, carrier-mediated distribution into tissues, and renal tubular reabsorption (4). Free carnitine is filtered by renal glomeruli and is 97% reabsorbed by the kidney tubules (7). Earlier studies suggest that malnourished children had lower serum carnitine, particularly among those with severe acute malnutrition (3, 5).


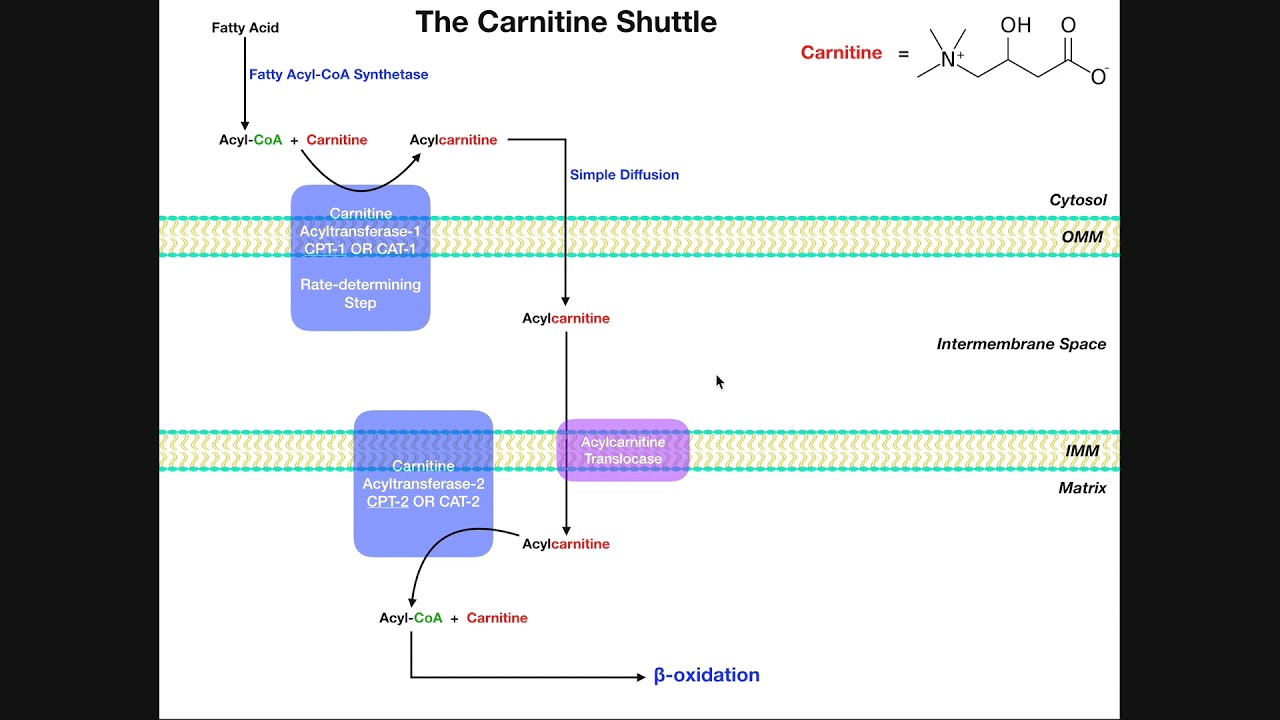


**(Source URL:** [**https://www.youtube.com/watch?v=C-k_Ehv0jbs**](https://www.youtube.com/watch?v=C-k_Ehv0jbs)**)**

**Figure 1: The Carnitine Shuttle**

Globally the rates of severe acute malnutrition (SAM) remain alarming and an estimated 14.3 million under-5 children are severely malnourished (8). Malnutrition is an underlying cause of nearly half of global deaths in under 5 children despite standardized rehabilitation protocols (8-10). It is associated with high relapse rates following discharge. The management of SAM occupies a unique position between clinical medicine and public health, and its management requires special attention (11). Recent trend shows that two-thirds of the children with SAM live in Asian countries including Bangladesh (8). The latest national survey of Bangladesh (BDHS 2017-18) shows that 31% of under-5 children were stunted (height for length z score), 9% were severely stunted, 22% were underweight (WAZ), 4% were severely underweight, 8% were wasted (Weight for Length z score) and 2% were severely wasted (12). The figures demonstrate that the burden of childhood malnutrition is still substantial in Bangladesh. It is reported earlier that malnourished children suffer from deficiencies of several essential nutrients, and carnitine is one of those essential nutrients. Past evidence also suggests that malnourished children suffer from carnitine deficiency, and it may afflict the growth and development of a children of growing age.

Carnitine deficiency may occur due to genetically determined metabolic disorders, acquired medical conditions, and some other external factors such as inadequate intake of carnitine, and decreased biosynthesis of carnitine (13-16). It is noted that the main features of carnitine deficiency are similar to the manifestation of mitochondrial insufficiency, for example, fatigue, muscle weakness, and hypotonia, slackness, drowsiness or irritability physical retardation, failure to thrive, recurrent infection, cardiovascular disorder, hypoglycemia, hyper ammonia, decrease school performance, etc. (17). Few studies showed carnitine has a role in the rate of weight gain in malnourished children (5). A positive correlation between albumin and plasma carnitine levels in PEM has also been reported (5). An incremental growth was observed in 22 out of 33 carnitine-administered patients who presented with failure to thrive (17) and this was attributed to the role of carnitine as a muscle growth factor. Carnitine helps in energy production in the form of ATP. Several studies found that ATP has beneficial effects on nutritional status (18). Therefore, it is assumed that carnitine may help in increasing weight gain by increasing the ATP production.

Recent evidence confirms that Environmental Enteric Dysfunction (EED), an asymptomatic small intestinal disorder, is characterized by secondary carnitine deficiency in children aged <60 months (19). EED is also characterized by small intestinal inflammation and abnormal gut permeability due to generalized disturbances of small intestinal structure and function. The histopathological features of EED includes blunting or atrophy of intestinal villi, increased inflammatory infiltrates in lamina propria, and hyperplasia of small intestinal crypts (20, 21). However, a number of fecal and blood biomarkers have been proposed to determine EED in low-income settings, for example, MPO, NEO, A1AT, KT ratio, and Citrulline. EED is an acquired condition and caused by repeated enteropathogen infections which damage the gut structure and results in impaired nutrient absorption (22, 23). This sub-clinical condition is pervasive in the under five children living in tropical countries. EED is found to be an emerging contributor of childhood growth faltering (24). However, the exact pathogenesis and metabolic consequences of EED are not well understood. Recent documents linking between EED and carnitine deficiency provides signals regarding role of carnitine deficiency and EED in childhood malnutrition. It is assumed that carnitine deficiency leading to EED may negatively affect the physical growth and cognitive development of young children (19). It is also possible that consequences of severe malnutrition can be perilous in young children if carnitine deficiency is coupled with EED (19). Nevertheless, evidence of carnitine status in diarrheal children with SAM is very limited in Bangladesh (5). The role of treatment of SAM in the improvement of carnitine status among malnourished children is not well known (5). As well as EED is pervasively common among children in the country. There may have specific biological obstacles concerning EED which render nutritional therapy ineffective or partially effective in children with SAM. The impact of L-carnitine supplementation on biomarkers of EED among malnourished children is also unknown, albeit EED can be ameliorated by L-carnitine supplementation as it is linked to carnitine deficiency. Therefore, we are proposing this study to assess carnitine level and explore its role on the rate of weight gain (anthropometric measurements), duration of hospital stays, and biomarkers of EED in severely malnourished children in Bangladesh.

## Research Design and Methods

| Describe the research design and methods and procedures to be used in achieving the specific aims of the research project. If applicable, mention the type of personal protective equipment (PPE), use of aerosol confinement, and the need for the use BSL2 or BSL3 laboratory for different part of the intended research in the methods.. Define the study population with inclusion and exclusion criteria, the sampling design, list the important outcome and exposure variables, describe the data collection methods/tools, and include any follow-up plans if applicable. Justify the scientific validity of the methodological approach (biomedical, social, gender, or environmental).  Also, discuss the limitations and difficulties of the proposed procedures and sufficiently justify the use of them. |
| --- |

**Study Design**

This study will be a double-blinded, placebo-controlled randomized clinical trial where the children in intervention arm will receive L-carnitine supplementation for 15 days. The control arm will receive placebo for the same duration. We will enroll diarrheal children with SAM both in intervention and control groups. In this study, SAM would be considered if WLZ/WHZ is <-3 SD of WHO child growth standards or clinical signs of bilateral pedal edema are present, or the mid-upper arm circumference (MUAC) is <115 mm.

**Study Site and population**

This study will be done in the Dhaka Hospital at icddr,b. This is the largest diarrheal disease hospital in the world. According to recent data, approximately 152,000 patients of all ages are treated here in a year (25). Among the treated patients, 62% are under the age of 5 years (25). In this study, we will enroll diarrheal children with SAM aged 9-24 months of both gender attending a longer stay unit (LSU).

**Study Duration:** 24 months

**Eligibility Criteria**

Inclusion criteria

- Diarrheal children with SAM aged 9-24 months
- Signed informed consent by the guardian/caregivers

Exclusion criteria

- Severe sepsis or Septic shock
- Patients already taking medications containing L- carnitine
- Children with Tuberculosis
- Children with congenital defects or chromosomal anomalies
- Children with a diagnosed case of Thalassemia
- Children with an active or previous history of convulsion

**Intervention**

Investigational products:

- L- carnitine oral solution (100mg/ml) for Group A
- Placebo for Group B- Oral solution identical in appearance and taste to the active preparation (L-carnitine syrup) with no therapeutic value

**Dosage:** 100 mg/kg/day, divided into 3 doses per day for 15 days.

**Packaging and labeling:** The investigational product will be dispensed in a 100ml bottle.

**Drug accountability**

The L-carnitine syrup formulation will be provided for 15 days to study participant at nutritional rehabilitation unit (NRU) under controlled set-up. They will be evaluated by measuring the rate of weight gain at 15 days of supplementation and 180 days of completion of supplementation. A telephonic follow-up will be done on after 15 days of completion of supplementation. Study investigator will update accountability records of the investigational product according to ICH E6, GCP guideline. Investigational product will not be provided to any third party.

**Workflow for enrolment:**

**
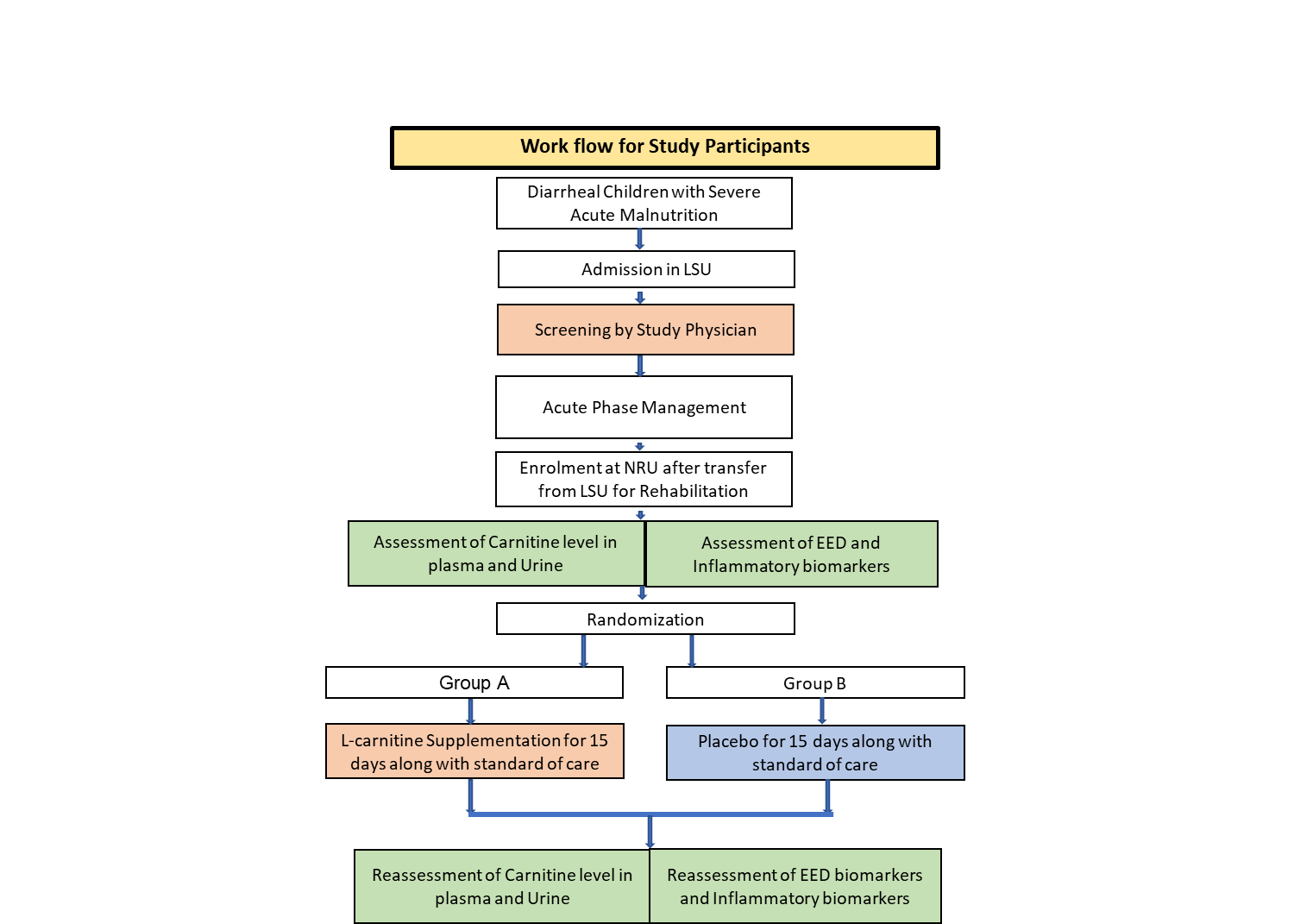
**

**Figure 2. Work flow for the participants enrolled in the study**

**Screening, Consenting and Baseline Data Collection**

The research staff will screen all the participants within the defined age groups according to the eligibility criteria. Participants fulfilling the eligibility criteria will be brought to study physician for clinical assessment. Upon signing a written informed consent, the participant will be enrolled by the study physician. Case record form (CRF) will be used to collect relevant information such as medical history including nature and duration of illness, medication for current illness; socio-demographic characteristics such as age, sex, religion, parental age with education, parent’s occupation, monthly family income, number of siblings etc. Information will also be collected about child’s feeding practice such as- history of breast feeding, formula or other complementary feeding, immunisation status, family history of tuberculosis, recent respiratory tract infection of any family members and past history of child’s pneumonia would be recorded.

**Randomization:**

In this proposed study we will use block randomization in following method:

- We will randomize the study participant in a way so that balance can be maintained across the treatment group and placebo group. Each “block” would have a specified number of randomly ordered treatment and placebo assignments.
- It will be a double blinded study. We both the investigators and study participants and their care givers won’t know who will be receiving the particular treatment.
- We have a plan to make 17 blocks- 8 blocks of size 4, 4 blocks of size 6, 4 blocks of size 8, 1 block of size 10.


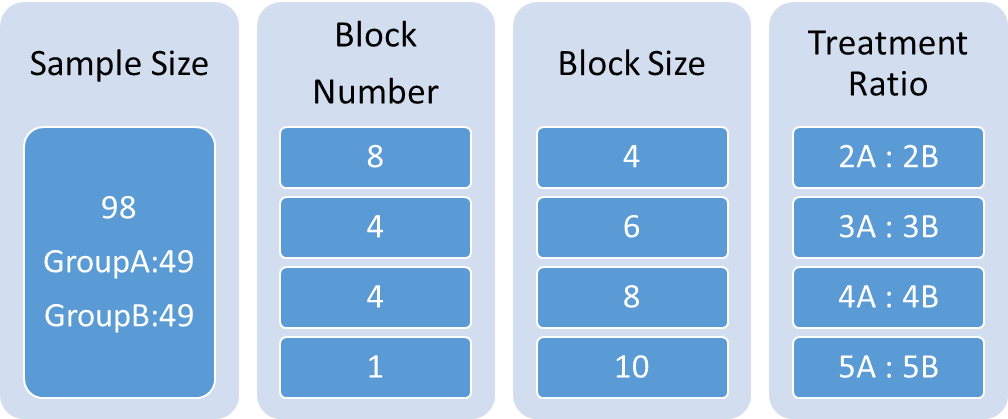


**Figure 3: Block Randomization**

| Criteria |  | Male | | Female | |
| --- | --- | --- | --- | --- | --- |
| WLZ score ≤3SD, or/and  MUAC <115 mm | Block Size 4 | AABB | BBAA | ABAB | BABA |
|  | Block Size 6 | AAABBB | BBBAAA | ABABAB | BABABA |
|  | Block Size 8 | AAAABBBB | BBBBAAAA | ABABABAB | BABABABA |
|  | Block Size 10 | ABABABABAB | | | |
| Bilateral Pedal Edema | Block Size 4 | AABB | BBAA | ABAB | BABA |

**Or,**

We will assign participants to the intervention or the control arm using permuted block randomization method with concealment to ensure that the allocation is not made before the participant has given their consent and joined the study. A random allocation sequence will be generated using a computerized random allocation system for permuted block randomization to ensure comparable allocation numbers at a certain equally spaced points in the sequence of patient assignment. A parallel type of randomization will be used. Reasonably large blocks with variable block sizes will be constructed to reduce the predictability. Random assignment will be prepared in advance by an independent researcher from icddr,b, who has no involvement with the trial.

**Anthropometric measurements**

All the measurements (length, weight, MUAC) will be taken at enrolment and on each day when the participants will receive intervention/placebo. If not possible, a one-day window period will be used. End-line anthropometry data will be collected on the 15^th^ day of supplementation. In addition, we will measure anthropometry on the 180^th^ day after the completion of supplementation to observe the long-term effect of the intervention on the rate of weight gain. Trained staff will take all the measurements as per the standard operating procedures (SOPs) and keep records in standard CRFs. Every measurement will be taken twice and the average will be documented. The Seca weighing scale will be used for weight measurement and Seca length board will be used for length measurement. To ensure the quality of anthropometric data, 5% of the participants will be measured second time within 24 hours of each data collection. Additionally, quality control assessment will be organized monthly and inter-rater reliability will be calculated to ensure the anthropometric data quality. Steps will be taken to minimize the discomfort and inconvenience of the participants during the anthropometric measurement.

**Biological sample collection**

We will collect blood, stool and urine from the participants at enrolment and at the end of nutrition intervention. Overall, 5 ml of whole venous blood will be collected aseptically from each of the participants as per the SOPs. The blood biomarkers that will be tested in this study are L-carnitine, Citrulline, KT Ratio, C-reactive protein (CRP), and Alpha-1-acid glycoprotein (AGP). CRP is an acute phase protein and can be detected during infection. Increased level of AGP in serum indicates systemic tissue injury, inflammation and infection. Both Citrulline and KT Ratio indicate EED in children. Stool will be collected to investigate the concentrations of MPO, NEO and A1AT in the fecal samples. We will measure L-carnitine levels in the urine samples of each participants. All the assays will be done at icddr,b.

Laboratory Investigation

| **Investigations** | **Type of tests** |
| --- | --- |
| L carnitine in plasma and urine | Enzyme-linked immunosorbent assay (ELISA)* |
| Myeloperoxidase (MPO) | Enzyme-linked immunosorbent assay (ELISA)* |
| Neopterin (NEO) |
| Alpha-1 antitrypsin (AAT) |
| Citrulline |
| Kynurenine: Tryptophan (KT) Ratio |
| C-reactive protein (CRP) |
| Alpha-1 acid glycoprotein (AGP) |
| Serum creatinine (S. Cr) | icddr,b diagnostic laboratory |
| Potential Renal Solute Load (PRSL) |

*ELISA will be done at icddr,b laboratory

Outcome measures/variable:

Primary Outcome Variable:

1. Rate of weight gain

Secondary Outcome Variable:

1. Duration of hospital stays
2. EED biomarkers (MPO, NEO, A1AT, KT Ratio, Citrulline)

## Sample Size Calculation and Outcome (Primary and Secondary) Variable(s)

| Clearly mention your assumptions. List the power and precision desired. Describe the optimal conditions to attain the sample size. Justify the sample size that is deemed sufficient to achieve the specific aims. |
| --- |

The sample size is estimated considering the primary outcome variable. For the primary objective, we have considered the study report, done by Alp Haiden et al, where they found the mean difference between the rate of weight gain after supplementation was 2.4 (The weight was measured in kg) and the standard deviation was 4.3 (5). Setting the level of confidence at 95% (Z1-α =1.645) and 80% of power, using the below formula, the estimated sample size is 49 participants in each group with a 10% attrition rate.

The sample size is calculated based on the following formula:

The total sample size for this study will be 98 (49 in Group A, and 49 in Group B). Sample size was estimated based on the following formula:

n= 2 × (
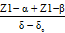
 ) ² × S²

Herein,

n = the sample size required in each group

S = standard deviation of the primary outcome variable = 4.3

δ = size of difference of clinical importance = 2.4

δ˳= clinically acceptable margin = 0.1

Z1-α = 1.645 (at a 5% level of significance)

Z1-β = 0.845 (for 80% power)

n = 49 participant in each group with 10% attrition rate

**Investigations**

The measurement of free L-carnitine in plasma will be done in every enrolled patient. Pre-Post test will be done. Pretest will be on the first day of enrolment and posttest will be on the 15^th^ day after completion of supplementation. Blood sample will be collected with proper aseptic procedure and SOP will be followed for blood L-carnitine level assay.

SOP for blood collection, processing and storage:

1. Blood collection site is prepared with a clean, disposable pad in a sterile fashion as to keep area contamination free.
2. Blood collection tube is prepared with appropriate label which include patient’s name, ID, date and time of collection.
3. Blood sample is collected in a race element free blood collection tube.
4. The blood tube is allowed to stand at room temperature for approximately 30 minutes.
5. After 30 minutes, tubes are centrifuged @3000 rpm for 10 minutes.
6. Plasma/serum are transferred into appropriately labeled cryo vials.
7. In a suitable time (as soon as possible) the samples are transferred to the central laboratory by maintaining cold chain.
8. Upon received in the lab, the samples are stored in the ultra-freezer (-80˚C) until analysis is complete.
9. After proving all results, samples will be discarded.

If centrifuge is not possible in the field site following steps need to be maintained for transferring whole blood to the central laboratory:

1. The blood tubes are allowed to stand at room temperature for few minutes.
2. The tubes are placed in a cool box with sample rack.
3. Sufficient Ice packs are needed to be placed inside the cool box. However it should be noted that ice pack should not touch the blood collection tube.
4. When blood collection is complete, an appropriate list for the entire specimen is prepared and this list is placed into the plastic bag. This plastic bag containing the completed specimen list is placed on top of the inner side of the lid before closing the cool box.
5. Samples are transferred to the central laboratory (icddr,b) as soon as possible (within 3-4 hours) by maintaining cold chain and if possible, temperature is monitored using thermometer.

To reduce the contamination, following things also needs to consider:

1. As much as possible, only certified trace element free supplies need to be used.
2. Powder-free gloves need be used during sample collection, preparation, storage and analysis.  Powdered gloves will contaminate sample.
3. All screening supplies, which include gauze pads, band aids, tissue paper, alcohol pads, and collection vials, need to be stored in a clean plastic box with a snap lid for storage.

**Experimental procedure for analysis of L-carnitine**

Plasma L-carnitine will be measured using Enzyme-linked immunosorbent assay (ELISA). [Company name: BIOMATIK; Catalog Number: EKC34382]

For internal quality control Bi-Level trace elements serum toxicology control, Normal range and High range (UTAK Laboratories Inc, CA 91355, USA) are used. These control materials are used in each day at the beginning of analysis to check both accuracy and precision. For ensuring the quality of results Immunobiology, Nutrition and Toxicology Laboratory also participate in College of American Pathologists (CAP), (External Quality assurance programs). [Reference: AOAC Official Methods of Analysis (1990) 15thed. 2nd Supplement (1991) p: 81]

**Management**

Children will be given their first dose of supplementation when they would be considered improved, i.e. no diarrhea for last 24 hours, patient fulfill the criteria of NRU transfer (presence of oedema, or/and WLZ <-3SD, or/and WAZ <-4SD). At NRU after randomization trained research staff will observe each child for 60 minutes following ingestion of investigational product and record any adverse event- nausea, vomiting etc. Vomiting is defined as the forceful emptying of stomach contents and will be recorded if it happened within 60 minutes following the administration of investigational product. Children will get supplementation of investigational product in addition to standard of care at NRU for 15 days. Supplementation will be given at every 8 hours under supervision of a trained staff.

**Study Days**Patients will be followed up every day by a study physician at NRU for 15 days. Adverse events will be documented and drug accountability recorded. During this intervention period if study participants fulfill the discharge criteria of NRU (WLZ/WHZ ≥ -2SD, WAZ ≥ -3SD and oedema resolved), we will discharge the patient with remaining dose of the syrup and advise them to come for follow up into after completion of the remaining dose. This same method will be applicable for both the intervention arm and placebo arm.

**Data processing Procedure**

- - Questionnaires will be visually scanned soon after the interview and marked for omissions, inconsistencies, or mistakes that will be addressed immediately
  - SPSS will be used and data will be entered after creating a template for each data entry file with appropriate logical and consistency checks
  - Data will be continuously entered as the data are being generated in the hospital
  - Data will be validated by a series of logical and range checks, producing summary statistics and tables
  - Data will be immediately copied on the hard disks of two computers as soon as data verification will be completed

**Safety Plan**

Gastrointestinal symptoms (Nausea, Vomiting, Diarrhea and Fever) are the most likely adverse event. The safety population will include all subjects who received at least one dose of the investigational products under study. Descriptive statistics will be used to summarize adverse events, safety results, and demographic variables. Reasons for dropouts and timing will be documented. Any dosing errors will be described, with any adverse events resulting.

## Data Analysis

Data will be entered into the pre-tested case record forms (CRFs) using SPSS (20.0 version, Armonk, NY) and finally cleaned with a repeated check. Data will be presented using frequency with percentage for categorical variables. Mean with standard deviation will be used for symmetrical continuous variables. Median with interquartile range will be used for asymmetrical numeric variables. To know the outcome of the intervention in our study children bivariate, crude analyses of the association will be done that will involve Chi-square or Fisher’s exact test for comparing differences in proportion and t-tests for numeric variables between the groups. Nnonparametric continuous data will be analyzed by Mann-Whitney U-test. Results from all children will be included in the analysis of the study on an intention-to-treat basis. Data from children withdrawn because of failure to respond, or voluntary dropouts will be included in the analysis up to the time of withdrawal. A supplementary analysis excluding the children withdrawn may also be done. A probability of less than 0.05 will be considered statistically significant.

## Data Safety Monitoring Plan (DSMP)

Data safety Monitoring will be rigorously performed throughout the study. All forms will be reviewed again by the study physician after enrolment and data collection followed by a supervisor for completeness, legibility, and internal consistency. A supervisor will perform random checks to ensure the validity of collected data by the study physician. The study physician will be trained accordingly for patient screening; enrolment, data collection, and study participants follow-up before initiation of the study according to rigorously standardized operating procedures (SOPs).

The study will be performed in compliance with the ‘Declaration of Helsinki’ (2000), the International Council of Harmonization (ICH), Tripartite Guidelines, Guideline for Good Clinical Practice (GCP). These procedures ensure the protection of the rights and the integrity of the study participants, adequate and correct conduct of all study procedures, adequate data collection, adequate documentation, and adequate data verification.

## Ethical Assurance for Protection of Human rights

The study protocol and data collection tools, and the informed consent forms to be used in the study will be submitted to the Research Review Committee (RRC) and the Ethical Review Committee (ERC) of icddr, b, and the study would be initiated only after receiving approval of the ERC. Before enrollment signed informed consent will be obtained from the parents/guardians of the children. The risk of enrolled subjects from participation in the study is minimal. Administration of L-carnitine and placebo supplements will be done with informed written consent. Patients will receive routine medical care. Adverse medical events are not anticipated from the routine procedures involved in the study. However, if any adverse events or injuries occur, providers will manage such events according to routine practice. The Ethical Review Committee of icddr, b will be notified of any serious adverse events (death, any life-threatening condition that requires ventilator/oxygen support, permanent damage or disability) and deviations from the protocol. Moreover, participation in this study is voluntary. Refusal to take part in the study will involve no breach in the continuity of care and the child will receive the standard treatment of this hospital. The study participants have the right to withdraw at any time from the study and also have the right to refuse to answer any question. Privacy, anonymity, and confidentiality of data/information identifying the child will strictly be maintained. We would keep all medical information, description of treatment, and results of the laboratory tests performed confidential, under lock and key, and none other than our research staff will have an access to this information.

**Timeline**

|  | ***Study Timeline***  (October'2021 - September'2022) | | | | | | |  |  |  |  |  |  |
| --- | --- | --- | --- | --- | --- | --- | --- | --- | --- | --- | --- | --- | --- |
| **Study Proceedings** | Oct | | Nov | Dec | Jan | Feb | Mar | Apr | May | June | July | Aug | Sep |
| Staff recruitment, training and orientation |  |  |  |  |  |  |  |  |  |  |  |  |  |
| Patient enrollment and data collection |  |  |  |  |  |  |  |  |  |  |  |  |  |
| Intervention |  |  |  |  |  |  |  |  |  |  |  |  |  |
| Laboratory Assessment |  |  |  |  |  |  |  |  |  |  |  |  |  |
| Data entry, cleaning and data preparation for analysis |  | |  |  |  |  |  |  |  |  |  |  |  |
| Data analysis and interpretations |  | |  |  |  |  |  |  |  |  |  |  |  |
| Report writing and preparation of manuscript |  | |  |  |  |  |  |  |  |  |  |  |  |

## Use of Animals

| Not applicable |
| --- |

##

## Collaborative Arrangements

| Not applicable. |
| --- |

## Facilities Available

The study will be conducted at the Dhaka Hospital of the International Centre for Diarrhoeal Disease Research, Bangladesh (icddr,b). This hospital is located in Dhaka city, the capital of Bangladesh. The vast majority of the patients come from poor socio-economic backgrounds from urban and peri-urban Dhaka, Bangladesh. This hospital has a separate ward including a nutrition rehabilitation ward, well-equipped laboratory facilities capable of performing all the clinical tests, and also has an Intensive Care Unit (ICU), equipped with mechanical ventilation and other facilities for the management of a critically ill patient. The hospital has a long track record of conducting clinical trials and other studies, some of which are known globally for contributing to improved health and nutrition of infants and young children.

**Anticipated results/outputs**

This study will facilitate us to evaluate the role of carnitine supplementation in the rate of weight gain and duration of hospital stay in children who are severely malnourished and received inpatient treatment. The knowledge on carnitine status as well as the prevalence of carnitine deficiencies at enrolment in children with severe acute malnutrition will help us to design a better treatment strategy in this vulnerable group of population.

**Potential Impact**

In addition to standard of care judicial use of the L-carnitine oral solution will be recommended if the study shows that L-carnitine supplementation increases the rate of weight gain and also affects the duration of hospital stay as well as EED biomarkers of SAM children.

## Literature Cited

|  |
| --- |

# Budget

**Detailed Budget for the study titled:** Role of L-Carnitine supplementation on rate of weight gain and biomarkers of Environmental Enteric Dysfunction (EED) in children with severe acute malnutrition

**Name of Principal Investigator:** Dr Jinat Alam

**Protocol Number:** PR-21046

**Division:** Nutrition and Clinical Services Division (NCSD)

**Funding Source:** icddr,b (Rainy Day Fund)

**Budget:** Direct: US$ 25,000; Indirect: 00% US$; Total: US$ 25,000

**Study period:**  From: October'21 to September'22

**Strategic Priority Code(s):**

| **Project Title:** | | Role of L-Carnitine supplementation on rate of weight gain and biomarkers of  Environmental Enteric Dysfunction (EED) in children with severe acute malnutrition | | | | | | | |  | |
| --- | --- | --- | --- | --- | --- | --- | --- | --- | --- | --- | --- |
| **Donor Name:** | | icddr,b | | | | | | | |  | |
| **Budget Period:** | | October'21 - September'22 | | | | | | | |  | |
| **PI Name and Email:** | | Dr. Jinat Alam, jinat.alam@icddrb.org | | | | | | | |  | |
| **Total Budget** | | 25,000 | |  | | | | | |  | |
|  | **Cost Categories** | | **Grade/ Step** | **Effort**  **(%)** | **No of**  **Staff** | **No. of months** | **Rate/m onth ($)** | **Year-1** | **Year-2** | | **Total**  **2021 to**  **2022**  **USD** |
|  |  |  |  |  |  |  |  | **Oct to**  **Dec 2021**  **USD** | **Jan to**  **Sep 2022**  **USD** | |  |
|  | **Personnel** | | | |  |  | |  |  | |  |
|  | Dr Jinat Alam (PI) | | Project Research Physician (Unclassified) | 50% | 1 | 8 | 702 | 1,053 | 1,931 | | 2,984 |
|  | Field Research Assistant | | GS3 (CSA) | 100% | 1 | 12 | 469 | 1,407 | 4,643 | | 6,050 |
|  | Health Worker/Field Attendant | | GS1 (CSA) | 100% | 1 | 12 | 353 | 1,059 | 3,495 | | 4,554 |
|  | **Sub-total** | | | |  |  |  | **3,519** | **10,068** | | **13,587** |
|  | **Equipment, Material and Supplies** | | | |  | **Qty** | **Rate** |  |  | |  |
|  | Supplies (Stock and Non-Stock items) | | | |  |  |  | 200 | 100 | | 300 |
|  | Laboratory Supplies (Reagents and Kits for testing L-carnitine and biomarkers of EED) | | | |  |  |  | 5,000 | 5,000 | | 10,000 |
|  | **Sub-total** | | | |  |  |  | **5,200** | **5,100** | | **10,300** |
|  | **Printing & Publications** | | | |  | **Qty** | **Rate** |  |  | |  |
|  | Printing & Photocopy | | | |  |  |  | 23 | 40 | | 63 |
|  | **Sub-total** | | | |  |  |  | **23** | **40** | | **63** |
|  | **Other Direct Costs** | | | | **Unit** | **Qty** | **Rate** |  |  | |  |
|  | Intervention Drug and Placebo | | | |  |  |  | 150 |  | | 150 |
|  | Laboratory & diagnostic tests, drugs and treatment of children with SAM | | | |  |  |  | 450 | 450 | | 900 |
|  | **Sub-total** | | | |  |  |  | **600** | **450** | | **1,050** |
|  |  | | | |  |  | |  |  | |  |
|  | **Total Direct costs** | | | |  |  | | **9,342** | **15,658** | | **25,000** |
|  | **IndirectCosts/Overhead** | | | |  |  | | - | - | | - |
|  | **Total Costs** | | | |  |  | | **9,342** | **15,658** | | **25,000** |

**Budget Justifications:**

| **Project Title:** | Role of L-Carnitine supplementation on rate of weight gain and biomarkers of Environmental Enteric Dysfunction (EED) in children with severe acute malnutrition |
| --- | --- |
| **Donor Name:** | icddr,b |
| **Budget Period:** | October'21 - September'22 |
| **PI Name and Email:** | Dr. Jinat Alam, jinat.alam@icddrb.org |
| **Budget elements** | **Justification** |
| **Personnel** | The proposed study activities include screening and enrollment of study participants, data collection, anthropometry, physical follow-ups and laboratory assays of enrolled participants. A substantial portion of the costs (Total $13,587) will be incurred for salary purpose as well as economic expenses of PI and study staff. |
| **Equipment, Material and Supplies** | A total cost of $10,300 will be incurred for purchasing study materials as well as laboratory kits and reagents. |
| **Printing & Publications** | A total cost of $63 will cover the photocopy of study materials |
| **Other Direct Costs** | A substantial amount of total $1,050 will be incurred for laboratory and diagnostic tests of the children, and for purchasing intervention drugs and placebo. |
| **Indirect Costs/Overhead** | **0% overhead cost** |

#

# Other Support

| Not Applicable. |
| --- |

**Appendix 1**

**External Reviewer: 1**

Page 1 of 2

**Title:** Role of L-Carnitine supplementation on rate of weight gain and biomarkers of Environmental Enteric Dysfunction (EED) in children with severe acute malnutrition

Summary of Referee’s Opinions: Please see the following table to evaluate the various aspects of the proposal by checking the appropriate boxes. Your detailed comments are sought on a separate, attached page.

|  | Rank Score | | |
| --- | --- | --- | --- |
|  | High | Medium | Low |
| Quality of project | X |  |  |
| Adequacy of project design | X |  |  |
| Suitability of methodology | X |  |  |
| Feasibility within time period | X |  |  |
| Appropriateness of budget | X |  |  |
| Potential value of field of knowledge | X |  |  |

## CONCLUSIONS

I support the application:

a) without qualification

X

b) with qualification

- on technical grounds

- on level of financial support

I do not support the application

Name of Referee: Jeffrey R. Donowitz

Signature: Date: 30 April, 2021

Position: Assistant Professor of Pediatric Infectious Diseases

Institution: Children’s Hospital of Richmond at Virginia Commonwealth University

Page 2 of 2

### Detailed Comments

Please briefly provide your opinions of this proposal, giving special attention to the originality and feasibility of the project, its potential for providing new knowledge and the justification of financial support sought; include suggestions for modifications (scientific or financial) where you feel they are justified. (Use additional pages if necessary)

**Title:** Role of L-Carnitine supplementation on rate of weight gain and biomarkers of Environmental Enteric Dysfunction (EED) in children with severe acute malnutrition

**PI:** Dr Jinat Alam

**Reviewer**: Jeffrey R. Donowitz, MD

Dr. Alam and colleagues are seeking to fill a crucial knowledge gap in the field of malnutrition and EED by performing a clinical trial of Levocarnitine for the treatment of severe acute malnutrition and EED. The trial is a well-designed randomized placebo-controlled double blinded trial. In addition block randomization will be used which may help to decrease bias given the smaller sample size. There is sufficient background data to suggest Levocarnitine’s utility in treating EED and malnutrition and thus, such a trial is scientifically warranted. The biomarkers of EED selected are broad, encompassing enteric inflammation, systemic inflammation, and intestinal integrity. The analysis plan is reasonable although authors should clearly state an adjusted model will also be created and describe the covariates included (or how they will be selected). The intervention is relatively benign when taken PO. The safety plan seems adequate for this small trial given the side effect profile of the intervention (i.e. generally do not see the cardiac effects when given PO).

Investigators plan to use a standard dose of 100mg/kg/day. However, literature supports titration to higher doses in certain children. It is unknown exactly how deficient children with SAM and EED are and thus it may be that this dose is insufficient to replete some children. Correction for baseline carnitine levels may be indicated in the final analysis but understanding the distribution of those baseline levels will also be a valuable addition to our current knowledge. In terms of the secondary outcome of study of Levocarnitine’s effect on EED, the interval of 15 days may be too short to recognize an effect on certain biomarkers. Specifically, those of gut permeability may take longer to correct. Also, the degree of baseline elevation in inflammatory biomarkers (including enteric inflammation) may be dependent on the type of pathogen causing the diarrhea that led to the child’s admission. Children with secretory diarrhea will have less elevation than those with inflammatory pathogens such as Campylobacter or Shigella. Inflammatory biomarkers to these pathogens may decrease regardless of intervention as the infection clears although likely will not normalize. One way to create a more heterogenous group without expensive pathogen testing would be to exclude children with fecal RBC positivity. Although pathogens such as Campylobacter are increasingly being recognized causing non-bloody diarrhea in addition to the more classic bloody diarrhea, this may help in excluding children with the highest levels of colitis (i.e. those that would lead to fecal RBC testing being positive). Alternatively, if the budget allows, fecal pathogen testing could be arranged and only children with viral disease enrolled as it would be expected that viral pathogens change the inflammatory biomarkers to a lesser degree. Of course, co-pathogen carriage may make screening difficult. Finally, there is evidence that the enteric microbiota plays an important role in carnitine metabolism. While likely beyond the scope of this project and budget, it would be reasonable to bank a separate aliquot of stool for the purpose of future microbiome studies. If this trial does demonstrate an effect in the treatment group, external funding for this line of question could potentially be secured. This should be a separate aliquot to limit freeze-thaw cycles and be stored in RNase/DNase free cryovials at -80^o^C.

Response to External Reviewer’s comments:

Reviewer: Jeffrey R. Donowitz, MD

Comment: “Dr. Alam and colleagues are seeking to fill a crucial knowledge gap in the field of malnutrition and EED by performing a clinical trial of Levocarnitine for the treatment of severe acute malnutrition and EED. The trial is a well-designed randomized placebo-controlled double blinded trial. In addition, block randomization will be used which may help to decrease bias given the smaller sample size. There is sufficient background data to suggest Levocarnitine’s utility in treating EED and malnutrition and thus, such a trial is scientifically warranted. The biomarkers of EED selected are broad, encompassing enteric inflammation, systemic inflammation, and intestinal integrity. The analysis plan is reasonable although authors should clearly state an adjusted model will also be created and describe the covariates included (or how they will be selected). The intervention is relatively benign when taken PO. The safety plan seems adequate for this small trial given the side effect profile of the intervention (i.e. generally do not see the cardiac effects when given PO).”

Response: Thank you very much for your comments and considerations.

Comment: “Investigators plan to use a standard dose of 100mg/kg/day. However, literature supports titration to higher doses in certain children.”

Response: Thank you so much for your opinion. We agree with you regarding evidence on titration. However, slow administration of the dose is also evident. Therefore, we will give the supplementation of oral solution slowly to our study participant as per guidance of published literature.

Comment: “It is unknown exactly how deficient children with SAM and EED are and thus it may be that this dose is insufficient to replete some children.”

Response: Dear reviewer, thank you for your valuable suggestion. In previous study on carnitine supplementation in SAM children they gave supplementation of 100 mg/kg/day and found significant weight gain in SAM children. Several studies also showed that supplementation of 50-150 mg/kg/day in 3-4 divided doses was effective in increasing the rate of weight gain. Therefore, we have designed our study to give 100mg/kg/day in 3 divided doses. We think it will be sufficient to replete the deficiency (5, 26).

Comment: “Correction for baseline carnitine levels may be indicated in the final analysis but understanding the distribution of those baseline levels will also be a valuable addition to our current knowledge.”

Response: Thank you sir for your valuable opinion. Yes, we will also try to understand the distribution of baseline carnitine level.

Comment: “In terms of the secondary outcome of study of Levocarnitine’s effect on EED, the interval of 15 days may be too short to recognize an effect on certain biomarkers. Specifically, those of gut permeability may take longer to correct.”

Response: Thank you sir for your valuable guidance and suggestion. In our study design we have a plan to collect sample for EED biomarkers for reassessment at day 15, after completion of intervention. We don’t know whether 15 days or 30 days are adequate to test faecal biomarkers. There is no data on the effect of carnitine supplementation on EED biomarkers. If we wait for 30 days to collect the samples, there would be also a chance of increasing the drop out rate. Because most of the caregivers would not allow their children to stay for such a long time in hospital. It is not feasible as well to visit their homes to collect biological samples. Therefore, we will collect samples and measure EED biomarkers at the end of intervention. Since it is the first study of its kind, we would find evidence whether 15 days are adequate or not.

Comment: “Also, the degree of baseline elevation in inflammatory biomarkers (including enteric inflammation) may be dependent on the type of pathogen causing the diarrhea that led to the child’s admission. Children with secretory diarrhea will have less elevation than those with inflammatory pathogens such as Campylobacter or Shigella. Inflammatory biomarkers to these pathogens may decrease regardless of intervention as the infection clears although likely will not normalize. One way to create a more heterogenous group without expensive pathogen testing would be to exclude children with fecal RBC positivity. Although pathogens such as Campylobacter are increasingly being recognized causing non-bloody diarrhea in addition to the more classic bloody diarrhea, this may help in excluding children with the highest levels of colitis (i.e. those that would lead to fecal RBC testing being positive). Alternatively, if the budget allows, fecal pathogen testing could be arranged and only children with viral disease enrolled as it would be expected that viral pathogens change the inflammatory biomarkers to a lesser degree. Of course, co-pathogen carriage may make screening difficult.”

Response: Thank you sir for your valuable advice. We will enrol the children and collect the base line samples when the acute phase will be managed and the study participants will be shifted to NRU for rehabilitation. We anticipate that diarrhoea would be resolved during the time of enrolment. Hence, we expect that the role of pathogens would be subsided. Moreover, our budget would not allow us to collect additional samples and perform culture and occult blood test. Therefore, we will not do culture or occult blood test in this study.

Comment: “Finally, there is evidence that the enteric microbiota plays an important role in carnitine metabolism. While likely beyond the scope of this project and budget, it would be reasonable to bank a separate aliquot of stool for the purpose of future microbiome studies. If this trial does demonstrate an effect in the treatment group, external funding for this line of question could potentially be secured. This should be a separate aliquot to limit freeze-thaw cycles and be stored in RNase/DNase free cryovials at -80^o^C.”

Response: Thank you sir for your valuable suggestion. We included the idea in our plan, and we will preserve the sample for future microbiome studies. (Please see page- 6)

External Reviewer: 2

Page 1 of 2

**Title:** Role of L-Carnitine supplementation on rate of weight gain and biomarkers of Environmental Enteric Dysfunction (EED) in children with severe acute malnutrition

Summary of Referee’s Opinions: Please see the following table to evaluate the various aspects of the proposal by checking the appropriate boxes. Your detailed comments are sought on a separate, attached page.

|  | Rank Score | | |
| --- | --- | --- | --- |
|  | High | Medium | Low |
| Quality of project |  | √ |  |
| Adequacy of project design | √ |  |  |
| Suitability of methodology | √ |  |  |
| Feasibility within time period | √ |  |  |
| Appropriateness of budget | No idea |  |  |
| Potential value of field of knowledge |  | √ |  |

## CONCLUSIONS

I support the application:

a) without qualification

√

b) with qualification

- on technical grounds

- on level of financial support

I do not support the application

Name of Referee: Dr. Md Mahbubul Hoque

Signature:

Date: 04.05.2021

Position: Professor of Paediatrics

Institution: Dhaka Shishu (Children) Hospital and Bangladesh Institute of Child Health

Page 2 of 2

### Detailed Comments

Please briefly provide your opinions of this proposal, giving special attention to the originality and feasibility of the project, its potential for providing new knowledge and the justification of financial support sought; include suggestions for modifications (scientific or financial) where you feel they are justified. (Use additional pages if necessary)

**Title:** Role of L-Carnitine supplementation on rate of weight gain and biomarkers of Environmental Enteric Dysfunction (EED) in children with severe acute malnutrition

**PI:** Dr Jinat Alam

**Reviewer**: Dr. Md Mahbubul Hoque

Some observation-

How L carnitine may help in increasing weight gain in SAM has not been well explained in background.

L carnitine helps to burn fatty acid within cell to produce energy, with this idea some studies done in adult to see the impact of L carnitine ingestion in reducing the wt. But in this study you want to see the impact on weight gain, so more explanation in this regards is necessary.

How dosage is calculated **(**100 mg/kg/day) for SAM or Infant is not mentioned?

My overall opinion is that this research can be done as at the end it can give us some knowledge on improvement of SAM with diarrhea management.

Thanks.

Response to External Reviewer’s comments:

Reviewer: Dr. Md Mahbubul Hoque

Comment: “How L carnitine may help in increasing weight gain in SAM has not been well explained in background.”

Response: Thank you very much for your comment. We have added a new paragraph on how L-carnitine supplementation may help in increasing weight gain in SAM children (Please see page 14).

Comment: “L carnitine helps to burn fatty acid within cell to produce energy, with this idea some studies done in adult to see the impact of L carnitine ingestion in reducing the wt. But in this study you want to see the impact on weight gain, so more explanation in this regards is necessary.”

Response: Thank you sir for your valuable opinion. We have added one paragraph with evidence about the impact of L-carnitine supplementation on weight gaining (Please see page 14).

Comment: “How dosage is calculated (100 mg/kg/day) for SAM or Infant is not mentioned?”

Response: The recommended dose of carnitine supplementation for children is 50-150 mg/kg/day and not more than 3 g/day). Besides, *Alp Haiden et al.* did a study on carnitine supplementation for SAM children at a dose of 100mg /kg/day. They found significant weight gain in children with SAM administering this dose (5). Hence, we have decided to give 100 mg/kg/day for our study participants (Diarrheal children with severe acute malnutrition).

Comment: “My overall opinion is that this research can be done as at the end it can give us some knowledge on improvement of SAM with diarrhea management.”

Response: Thank you very much for your valuable comments and consideration.

**Appendix 2**

| Protocol No: 21046 | Version No: 1.00 | Date: 24.05.2021 |
| --- | --- | --- |

**Gender Analysis Tool:**

| **In Relation to Levocarnitine syrup supplementation in young children** | **Are there sex differences in** | **How do biological differences between women and men influence their** | **How do the different roles and activities of men and women affect their** | **How do gender norms/values affect men and women’s** | **How do access to, and control over resources affect men and women’s** |
| --- | --- | --- | --- | --- | --- |
| **Vulnerability:**    **Incidence ****  **Prevalence ****  **(male/female)** | No such data are available for Levocarnitine syrup supplementation in children | No such data are available for Levocarnitine syrup supplementation in children | No such data are available for Levocarnitine syrup supplementation in children | No such data are available for Levocarnitine syrup supplementation in children | No such data are available for Levocarnitine syrup supplementation in children |
| **Health seeking behaviour** | No such data are available for Levocarnitine syrup supplementation in children | No such data are available for Levocarnitine syrup supplementation in children | No such data are available for Levocarnitine syrup supplementation in children | No such data are available for Levocarnitine syrup supplementation in children | No such data are available for Levocarnitine syrup supplementation in children |
| **Ability to access health services** | No such data are available for Levocarnitine syrup supplementation in children | No such data are available for Levocarnitine syrup supplementation in children | No such data are available for Levocarnitine syrup supplementation in children | No such data are available for Levocarnitine syrup supplementation in children | No such data are available for Levocarnitine syrup supplementation in children |
| **Experience with health services and health providers** | No such data are available for Levocarnitine syrup supplementation in children | No such data are available for Levocarnitine syrup supplementation in children | No such data are available for Levocarnitine syrup supplementation in children | No such data are available for Levocarnitine syrup supplementation in children | No such data are available for Levocarnitine syrup supplementation in children |
| **Preventive and Treatment options, responses to treatment or rehabilitation** | No such data are available for Levocarnitine syrup supplementation in children | No such data are available for Levocarnitine syrup supplementation in children | No such data are available for Levocarnitine syrup supplementation in children | No such data are available for Levocarnitine syrup supplementation in children | No such data are available for Levocarnitine syrup supplementation in children |
| **Outcome of health problem** | No such data are available for Levocarnitine syrup supplementation in children | No such data are available for Levocarnitine syrup supplementation in children | No such data are available for Levocarnitine syrup supplementation in children | No such data are available for Levocarnitine syrup supplementation in children | No such data are available for Levocarnitine syrup supplementation in children |
| **Consequences (economic& social, including attitudinal)** | No such data are available for Levocarnitine syrup supplementation in children | No such data are available for Levocarnitine syrup supplementation in children | No such data are available for Levocarnitine syrup supplementation in children | No such data are available for Levocarnitine syrup supplementation in children | No such data are available for Levocarnitine syrup supplementation in children |

**Biography of the Investigators**

1. **Name: Dr. Jinat Alam**
2. **Present Position:** Project Research Physician, NCSD, icddr,b
3. **Educational background**: (last degree and diploma & training relevant to the present research proposal)

|  | Institution | Year |
| --- | --- | --- |
| MBBS | Ibn Sina Medical College and Hospital (Dhaka University, Bangladesh) | 2014 |
| Training | Internship training on Medicine, Surgery, and Gynaecology in Ibn Sina Medical College and Hospital, Dhaka | 2015 |
| Training | Residential training in paediatrics, icddr,b | 2018-19 |
| Fellowship | Clinical Fellowship, icddr,b | 2017-19 |

1. **Ethics Certification:**

|  |  | If Yes | | |
| --- | --- | --- | --- | --- |
|  |  | Issuing Authority | Registration No | Done on |
| No | Yes | PHRP | 2819860 | Issued in 07April, 2019 |

**Note**: If the response is “no”, please get certification from CITI or NIH before study initiation and submit a copy to the Committee Coordination Secretariat

1. **List of ongoing research protocols/ activities**

| Protocol/ Activity Number | Role in the protocol/ activity (PI, Co-PI, Co-I) | Starting date | End date | Percentage of time |
| --- | --- | --- | --- | --- |
| N/A | - | - | - | - |

1. **Publications**

| Types of publications | Numbers |
| --- | --- |
| - Original scientific papers in peer-review journals | 0 |
| - Peer reviewed articles and book chapters | 0 |
| - Papers in conference proceedings | 0 |
| - Letters, editorials, annotations, and abstracts in peer-reviewed journals | 0 |
| - Working papers | 1 |
| - Monographs | 0 |

1. **Five recent publications including publications relevant to the present research protocol-**N/A

**Biography of the Investigators**

1. **Name: Dr Shah Mohammad Fahim**
2. **Present Position:** Research Investigator, Nutrition and Clinical Services Division, icddr,b
3. **Educational background**: (last degree and diploma & training relevant to the present research proposal)

|  | Institution | Year |
| --- | --- | --- |
| MPH | American International University- Bangladesh | 2018 |
| MBBS | Sher-E-Bangla Medical College, Barisal | 2013 |
| Training | Internship training on Medicine, Surgery, and Gynaecology in Sher-E-Bangla Medical College & Hospital, Barisal | 2014 |
| Training | Statistical training on analysing Real Life Data organized by icddr,b and Queensland University, Australia | 2018 |
| Training | Advanced statistical training on Biostatistics and R organized by icddr,b and Duke University, USA | 2018 |

1. **Ethics Certification:**

|  |  | If Yes | | |
| --- | --- | --- | --- | --- |
|  |  | Issuing Authority | Registration No | Valid Until |
| **No** | Yes | NIH | 2562242 | Issued on 16 November, 2017 |
| **No** | Yes | PHRP | 2826861 | Issued 22 June, 2019 |

**Note**: If the response is “no”, please get certification from CITI or NIH before study initiation and submit a copy to the Committee Coordination Secretariat

1. **List of ongoing research protocols/ activities**

| Protocol/ Activity Number | Role in the protocol/ activity (PI, Co-PI, Co-I) | Starting date | End date | Percentage of time |
| --- | --- | --- | --- | --- |
| PR-20051 | PI | 03.08.2020 | 02.08.2021 | 20% |
| PR-19082 | PI | 01.10.2019 | 30.09.2020 | 20% |
| PR-19036 | Co-PI | 09.07.2019 | 08.07.2020 | 10% |
| PR-19074 | Co-PI | 01.07.2019 | 30.06.2020 | 10% |
| PR-20022 | Co-I | 01.06.2020 | 30.05.2021 | 10% |
| PR-20023 | Co-PI | 16.08.2020 | 15.08.2021 | 10% |
| PR-20025 | Co-I | 01.07.2020 | 30.06.2021 | 10% |
| PR-20089 | Co-I | 30.11.2020 | 29.22.2021 | 10% |

1. **Publications**

| Types of publications | Numbers |
| --- | --- |
| - Original scientific papers in peer-review journals | 25 |
| - Peer reviewed articles and book chapters |  |
| - Papers in conference proceedings | 10 |
| - Letters, editorials, annotations, and abstracts in peer-reviewed journals | 0 |
| - Working papers | 1 |
| - Monographs |  |

1. **Five recent publications including publications relevant to the present research protocol**
   1. **Fahim SM**, Das S, Gazi MA, Alam MA, Hasan MM, Hossain MS, Mahfuz M, Rahman MM, Haque R, Sarker SA, Mazumder RN. Helicobacter pylori infection is associated with fecal biomarkers of environmental enteric dysfunction but not with the nutritional status of children living in Bangladesh. PLoS neglected tropical diseases. 2020 Apr 23;14(4):e0008243.
   2. **Fahim SM**, Das S, Gazi MA, Alam MA, Mahfuz M, Ahmed T. Evidence of gut enteropathy and factors associated with undernutrition among slum-dwelling adults in Bangladesh. The American Journal of Clinical Nutrition. 2020 Jan 7.
   3. Mahfuz M, Alam MA, Das S, **Fahim SM**, Hossain MS, Petri Jr WA, Ashorn P, Ashorn U, Ahmed T. Daily supplementation with egg, cow milk, and multiple micronutrients increases linear growth of young children with short stature. The Journal of nutrition. 2020 Feb 1;150(2):394-403.
   4. Mahfuz M, Murray-Kolb LE, Hasan SM, Das S, **Fahim SM**, Alam MA, Caulfield L, Ahmed T. Why Do Children in Slums Suffer from Anemia, Iron, Zinc, and Vitamin A Deficiency? Results from a Birth Cohort Study in Dhaka. Nutrients. 2019 Dec;11(12):3025.
   5. **Fahim SM**, Das S, Gazi MA, Mahfuz M, Ahmed T. Association of intestinal pathogens with faecal markers of environmental enteric dysfunction among slum‐dwelling children in the first 2 years of life in Bangladesh. Tropical Medicine & International Health. 2018 Nov;23(11):1242-50.

**Biography of the Investigators**

1. **Name: Dr Tahmeed Ahmed**
2. **Present Position:** Executive Director and Senior Director, Nutrition and Clinical Services Division, icddr,b and Professor of Public Health Nutrition, James P. Grant School of Public Health, BRAC University
3. **Educational background**: (last degree and diploma & training relevant to the present research proposal)

|  | Institution | Year |
| --- | --- | --- |
| PhD | University of Tsukuba, Japan | 1996 |
| MBBS | University of Dhaka | 1983 |
| Training | Clinical training in Pediatrics, University of Tsukuba Hospital | 1990-1992 |
| Training | Residential training in Pediatrics, Dhaka Child Hospital | 1989-1990 |

1. **Ethics Certification:**

|  |  | If Yes | | |
| --- | --- | --- | --- | --- |
|  |  | Issuing Authority | Registration No | Valid Until |
| **No** | Yes | NIH | 1933611 | Issued on 12 August 2015 |

**Note**: If the response is “no”, please get certification from CITI or NIH before study initiation and submit a copy to the Committee Coordination Secretariat

1. **List of ongoing research protocols/ activities**

| Protocol/ Activity Number | Role in the protocol/ activity (PI, Co-PI, Co-I) | Starting date | End date | Percentage of time |
| --- | --- | --- | --- | --- |
| The BEED study | PI | 16-11-2015 | 31-10-2020 | 15 |
| The Microbiota Directed Complementary Food Trials | PI | 28-11-2017 | 01-03-2021 | 15 |
| Bangladesh Longitudinal Investigation of Emerging Vascular and nonvascular Events – Rural (BELIEVE-Rural) study | Co-PI | 02-06-2019 | 31-10-2022 | 10 |
| The GMP study | Co-PI | 10-03-2018 | 31-12-2020 | 10 |
| Evaluation of SUCHANA | Co-PI | 01-12-2018 | 30-06- 2020 | 10 |
| Synbiotics for the Early Prevention of Severe Infections in Infants (SEPSIS) trial | Co-PI | 01-09-2019 | 31-12-2022 | 10 |
| A randomized, double-blind community trial of supplementation of varied doses of zinc in micronutrient powders in young, Bangladeshi children (ZIPT trial) | Co-PI | 01-03-2017 | 31-03-2020 | 10 |

1. **Publications**

| Types of publications | Numbers |
| --- | --- |
| a. Original scientific papers in peer-review journals | 370 |
| b. Peer reviewed articles and book chapters | 18 |
| c. Papers in conference proceedings | 25 |
| d. Letters, editorials, annotations, and abstracts in peer-reviewed journals | 5 |
| e. Working papers | 10 |
| f. Monographs | 1 |

1. **Five recent publications including publications relevant to the present research protocol**
   1. Chen RY, Mostafa I, Hibberd MC, Das S, Mahfuz M, Naila NN, Islam MM, Huq S, Alam MA, Zaman MU, Raman AS, **Ahmed T**. A Microbiota-Directed Food Intervention for Undernourished Children. New England Journal of Medicine. 2021 Apr 7.
   2. .Chen RY, Kung VL, Das S, Hossain MS, Hibberd MC, Guruge J, Mahfuz M, Begum SK, Rahman MM, Fahim SM, Gazi MA, **Ahmed T**. Duodenal microbiota in stunted undernourished children with enteropathy. New England Journal of Medicine. 2020 Jul 23;383(4):321-33.Effects of microbiota-directed foods in gnotobiotic animals and undernourished children. Science. 2019; 365(6449). pii: eaau4732. doi: 10.1126/science.aau4732. PubMed PMID: 31296738.
   3. Fahim SM, Das S, Gazi MA, Alam MA, Mahfuz M, **Ahmed T**. Evidence of gut enteropathy and factors associated with undernutrition among slum-dwelling adults in Bangladesh. The American Journal of Clinical Nutrition. 2020 Jan 7.
   4. Hossain MS, Begum SK, Rahman MM, Mazumder RN, Parvez M, Gazi MA, Hasan MM, Fahim SM, Das S, Mahfuz M, Sarker SA. Alterations in the histological features of the intestinal mucosa in malnourished adults of Bangladesh. Scientific Reports. 2021 Jan 27;11(1):1-8.
   5. Mahfuz M, Alam MA, Das S, Fahim SM, Hossain MS, Petri Jr WA, Ashorn P, Ashorn U, **Ahmed T**. Daily supplementation with egg, cow milk, and multiple micronutrients increases linear growth of young children with short stature. The Journal of nutrition. 2020 Feb 1;150(2):394-403.

**Biography of the Investigators**

- 1. **Name: Md Amran Gazi**
  2. **Present Position:** Assistant Scientist, Nutrition and Clinical Services Division, icddr,b
  3. **Educational background**:

|  | Institution | Year |
| --- | --- | --- |
| MS | University of Dhaka | 2013 |
| BS | University of Dhaka | 2012 |

- 1. **Ethics Certification:**

|  |  | If Yes | | |
| --- | --- | --- | --- | --- |
|  |  | Issuing Authority | Registration No | Valid Until |
| **No** | Yes | PHRP | 2826764 | Issued 22 june 2019 |

- 1. **List of ongoing research protocols/ activities**

| Protocol/ Activity Number | Role in the protocol/ activity (PI, Co-PI, Co-I) | Starting date | End date | Percentage of time |
| --- | --- | --- | --- | --- |
| PR-20025 | Co-PI | June 2020 | May 2021 |  |

- 1. **Publications**

| Types of publications | Numbers |
| --- | --- |
| Original scientific papers in peer-review journals | 23 |
| Peer reviewed articles and book chapters | 0 |
| Papers in conference proceedings | 3 |
| Letters, editorials, annotations, and abstracts in peer-reviewed journals | 0 |
| Working papers | 2 |
| Monographs | 0 |

- 1. **Five recent publications including publications relevant to the present research protocol**

1. MS Hossain, SMKN Begum, MM Rahman, RN Mazumder, M Parvez, **Gazi MA**, Ahmed T (2021). Alterations in the histological features of the intestinal mucosa in malnourished adults of Bangladesh. Scientific Reports
2. **Gazi MA***, Das S, Siddique MA‎, Fahim SM, Petri Jr. WA, Mahfuz M, Ahmed T (2020). Plasma kynurenine to tryptophan ratio is negatively associated with linear growth of children living in a slum of Bangladesh: results from a community-based intervention study. American journal of tropical medicine and hygiene
3. S Das, **MA Gazi**, MM Hasan, SM Fahim, MA Alam, MS Hossain, M Mahfuz, Ahmed T (2020). Changes in Retinol Binding Protein 4 Level in Undernourished Children After a Nutrition Intervention Are Positively Associated With Mother’s Weight but Negatively With Mother’s Height, Intake of Whole Milk, and Markers of Systemic Inflammation: Results From a Community-Based Intervention Study. Food and Nutrition Bulletin
4. **Gazi MA***, Mahmud S, Fahim SM, Islam MR, Das S, Mahfuz M, Ahmed T (2020). Questing function and structure of hypothetical proteins from *Campylobacter jejuni*: a computer aided approach. Bioscience reports
5. Fahim SM, Das S, **Gazi MA**, Mahfuz M, Ahmed T (2020). Evidence of gut enteropathy and determinants of undernutrition among slum-dwelling adults in Bangladesh. Am J Clin Nutr. 2020;111(3):657-666

**Biography of the Investigators**

1. **Name: Dr Md Ridwan Islam**
2. **Present Position:** Study Physician
3. **Educational background**: (last degree and diploma & training relevant to the present research proposal)

|  | Institution | Year |
| --- | --- | --- |
| MPH | North South University | Thesis ongoing |
| MBBS | Holy Family Red Crescent Medical College | 2013 |
| Training | Internship Training in Medicine, Surgery and Gynaecology | 2013-2014 |

1. **Ethics Certification:**

|  |  | If Yes | | |
| --- | --- | --- | --- | --- |
|  |  | Issuing Authority | Registration No | Valid Until |
| **No** | Yes | PHRP | 2802104 | Issued 25 December, 2018 |

**Note**: If the response is “no”, please get certification from CITI or NIH before study initiation and submit a copy to the Committee Coordination Secretariat

1. **List of ongoing research protocols/ activities**

| Protocol/ Activity Number | Role in the protocol/ activity (PI, Co-PI, Co-I) | Starting date | End date | Percentage of time |
| --- | --- | --- | --- | --- |
| PR-20023 | PI | 16 August 2020 | 15 August 2021 | 100% |

1. **Publications**

| Types of publications | Numbers |
| --- | --- |
| 1. Original scientific papers in peer-review journals | 2 |
| 2. Peer reviewed articles and book chapters | 0 |
| 3. Papers in conference proceedings | 1 |
| 4. Letters, editorials, annotations, and abstracts in peer-reviewed journals | 0 |
| 5. Working papers | 6 |
| 6. Monographs | 0 |

1. **Five recent publications including publications relevant to the present research protocol**
2. **Islam MR**, Nuzhat S, Fahim SM, Palit P, Flannery RL, Kyle DJ, Mahfuz M, Islam MM, Sarker SA, Ahmed T. Antibiotic exposure among young infants suffering from diarrhoea in Bangladesh. Journal of Paediatrics and Child Health. 2021 Mar;57(3):395-402.
3. Nuzhat S, Shahunja KM, Shahid AS, Khan SH, Islam SB, **Islam MR**, Ahmed T, Chisti MJ, Hossain MI, Faruque AS. Diarrhoeal children with concurrent severe wasting and stunting compared to severe wasting or severe stunting. Tropical Medicine & International Health. 2020 Aug;25(8):928-35.

Information Sheet for Parent / Legal Guardian of the Participant

Main Trial

| Protocol No: 21046 | Version No: 1.00 | Date: 24.05.2021 |
| --- | --- | --- |

Protocol Title: Role of L-Carnitine supplementation on rate of weight gain and biomarkers of Environmental Enteric Dysfunction (EED) in children with severe acute malnutrition

Principal Investigator’s name: Dr Jinat Alam

Organization: International Centre for Diarrhoeal Disease Research, Bangladesh

Purpose of the research

Background

Globally the rates of severe acute malnutrition (SAM) remain alarming and it is an underlying cause of nearly half of global deaths in under 5 children. Research states that malnourished children suffer from deficiencies of several essential nutrients. L-carnitine is one of the essential nutrients, which helps in energy production but lower in malnourished children. It is important for the improvement of heart and brain function, muscle movement, and many other body processes as well as disease prevention. There is another sub-clinical intestinal disorder named Environmental Enteric Dysfunction (EED), which can be persistent in the malnourished children living in tropical countries. Carnitine deficiency leading to EED may negatively affect the growth and development of young children. Oral Levocarnitine syrup could provide a new therapeutic addition for children with malnutrition and EED. This drug established a safety record in pediatrics while being used for the treatment of Down’s syndrome and heart disease.

We are conducting a study to evaluate the response to levocarnitine syrup in the pediatric patient population (9-24 months) with SAM and comparing the effectiveness with standard care of treatment. We also evaluate the effect of levocarnitine syrup in the management of EED.

Why invited to participate in the study?

We are inviting you for participation of your child in our study as he / she is suffering from SAM and aged 9-24 months. We are looking for children like him/her for our study. As your child has severe acute malnutrition and needs special facility-based management at the nutritional rehabilitation unit (NRU), we assume that adding syrup levocarnitine with WHO standard treatment of SAM will increase the rate of weight gain and decrease the duration of hospital stay as well as will also improve the condition of EED.

This information, if available, will help the doctors to add a new dimension to the management of SAM and EED. This is why we are requesting you to help us by giving your permission to include your child in our study.

Methods and procedures [What is expected from the participants of the research study?]

If you allow participation of your child in our study, you may expect the followings:

- The doctors, nurses and other staff of this hospital will provide the usual good care and treatment to your child. Participating in this study will not change the standard treatment of this hospital in any way, and the laboratory investigations if required for management of your child, will also be done according to the policy and guidelines of this hospital.
- We would ask you some questions related to your child’s illness, and perform thorough physical examinations on the enrolment day in the study and on each day of hospitalization to assess the progress of illness (improvement or deterioration).
- We would provide either levocarnitine syrup or placebo thrice daily for 15 days in addition to WHO standard care of treatment of malnutrition for 15 days. This study drugs are safe as mentioned earlier.
- We would collect urine, and stool samples of the baby on the day of enrolment and after completion of the 15^th^ day of supplementation and on 180^th^ day after completion of supplementation we will collect stool sample only.
- We would also collect 5 ml blood samples of the baby on the day of enrolment and after completion of the 15^th^ day of supplementation. Blood samples will be collected via the traditional method of the venipuncture (cubital vein) by needle and syringe.
- After completion of treatment, the doctors of this hospital will discharge your child from the NRU, according to the policy and guidelines of this hospital. What it means is that the management of your child and her/his discharge will not be influenced by the participation of your child in our study.
- Your co-operation for the study activities is very necessary.

Risk and benefits

Anticipated potential risks:

There is no major risk involved in the participation of your child in the study. Possible adverse events may be nausea, vomiting, diarrhea. Despite taking precautions, if your child develops any symptoms due to this study procedure, we would provide appropriate treatment at the Dhaka Hospital of icddr,b. Your child may experience little discomfort or pain while providing the blood samples. There is no major risk involved in giving blood samples.

Anticipated potential benefits:

Your infant will be directly and indirectly benefited from participating in the study. We are assuming due to L-carnitine deficiency, your child is suffering from severe acute malnutrition and EED. Through this study, you will be able to know the current status of L-carnitine and EED of your child. Moreover, your infant would be able to contribute to our understanding to develop more effective treatments for malnutrition and new treatment for EED. In the long term, the results of this study would benefit other children in Bangladesh and elsewhere by helping us understand the effects of levocarnitine syrup.

Privacy, anonymity and confidentiality

We do hereby state that privacy, anonymity and confidentiality of data/information identifying your child will strictly be maintained. We would keep all medical information, description of treatment, and results of the laboratory tests performed on your child confidential, under lock and key, and none other than our research staff will have an access to this information. No one other than this group of investigators, regulatory authorities and the Ethical Review Committee (a group of experts which protects the interest of study participants) of icddr,b and investigators sponsor of this study would have access to such information. Your child’s name and identity will not be disclosed while analysing or publishing the results of this study.

Future use of information

In the case of future use of the information collected from this study, privacy, anonymity and confidentiality of information will be maintained. We will store the stool, urine, and blood samples in a way that your child’s identity will not be recognized, and use the samples for performing tests that are modified in the near future for superior results, as well as new tests for studying the drug response. No further consent will be requested for such studies. The future use of the information collected through the study will not be of a commercial nature.

Right not to participate and withdraw

Your child’s participation in the study is voluntary, and you have the sole authority to decide for or against your patient’s participation. You would also be able to withdraw your child’s participation any time during the study, without showing any cause. If you decide to withdraw from the study after enrolment the samples/data that are already collected up to that point will be kept and used anonymously for future analysis. Refusal to take part in or withdraw from the study will involve no penalty or loss of care, benefits or attention.

Principle of compensation

Treatment at this hospital is free for all patients, and your child will not be an exception. Similarly, we will not pay money for participation in our study. If your child has a study-related injury s/he will receive standard care at the Dhaka Hospital (Cholera Hospital) in Mohakhali, Dhaka. If you need to come for follow up in to our hospital at day 15 and day 180 after completion of supplementation, we will only provide you the travel allowances.

Consent Form for Parent / Legal Guardian of the Participant

Main Trial

| Protocol No: 21046 | Version No: 1.00 | Date: 24.05.2021 |
| --- | --- | --- |

Protocol Title: Role of L-Carnitine supplementation on rate of weight gain and biomarkers of Environmental Enteric Dysfunction (EED) in children with severe acute malnutrition

Principal Investigator’s name: Dr Jinat Alam

Organization: International Centre for Diarrhoeal Disease Research, Bangladesh

If you agree to our proposal for enrolling your child in our study, please put √ mark on appropriate box (es) of the following and finally sign on the specified place for you:

I have read the information sheet for parent / legal guardian of the participants,

version 1.00, have had the opportunity to ask question, discuss the study,

and received satisfactory answers. Yes No

I understand that I am free to leave the study with my child without

giving any reason. Yes No

I agree to the collection of up to 5 ml of blood from my child. Yes No

I agree that anonymised blood samples of my child can be preserved

for future study. Yes No

I agree to the collection of stool/faecal material from my child. Yes No

I agree to the collection of urine sample from my child. Yes No

I understand that the information I give is confidential. Yes No

I agree to my child’s identifiable data being used for future studies. Yes No

I agree to being contacted in the future for studies related to this research. Yes No

I understand that relevant sections of my child’s medical notes and data

collected during the study may be looked at by individuals from the

sponsor and by regulatory authorities, where it is relevant to my child’s

taking part in this research. I give my permission for those individuals

to have access to my child’s records. Yes No

I give my consent to take participation of my child in the study. Yes No

__________________________________________ ______________________

Signature or left thumb impression of Date

Parent/ Guardian/ Attendant

_______________________________________ ____________________

Signature or left thumb impression of the witness Date

_______________________________________ ____________________

Signature of the PI or his/her representative Date

Communication:

If you have any question you can ask me right now or at any time later to the below mentioned personnel:

| Purpose of contact | Name and address | Address for communication |
| --- | --- | --- |
| For any question related to the study, or any problem | Dr Jinat Alam  Project Research Physician & Principal Investigator of the study | Address: Dhaka Hospital, icddr,b, Mohakhali, Dhaka-1212  Mobile: 01676742255 |
| To know the rights or benefits or to log any complain or dissatisfaction | M A Salam Khan (IRB Coordinator) | IRB Secretariat, Research Administration, icddr,b, Mohakhali, Dhaka-1212  Phone: (+88-02) 9827084 or Mobile: 01711428989 |

Thank you for your cooperation.

**অবহিতকরন পত্র (Main Trial)**

| Protocol No: 21046 | Version No: 1.00 | Date: 24.05.2021 |
| --- | --- | --- |

**গবেষণা প্রকল্পের শিরোনামঃ** Role of L-Carnitine supplementation on rate of weight gain and biomarkers of Environmental Enteric Dysfunction (EED) in children with severe acute malnutrition

**প্রধান গবেষকের নামঃ**- ডাঃ জিনাত আলম

**প্রতিষ্ঠানঃ** - আইসিডিডিআর,বি

**গবেষণার উদ্দেশ্যঃ**

**পটভুমিঃ**

বিশ্বব্যাপী মারাত্মক তীব্র অপুষ্টি (SAM) এর হার এখনও উদ্বেগজনক অবস্থানে বিরাজমান। পাঁচ বছরের কম বয়সী শিশুদের প্রায় অর্ধেকের মৃত্যুর অন্তর্নিহিত কারণ মারাত্মক তীব্র অপুষ্টি। গবেষণায় দেখা গেছে মারাত্মক তীব্র অপুষ্টিতে আক্রান্ত শিশুরা বিভিন্ন প্রয়োজনীয় শারীরিক উপাদানের ঘাটতিতে ভুগে থাকে। এল-কার্নিটিন, শরীরের একটি প্রয়োজনীয় পুষ্টি উপাদান যা শক্তি উৎপাদন এবং শরীরের অন্যান্য কাজে গুরুত্বপূর্ণ ভূমিকা পালন করে থাকে। এটি হার্ট, পেশী, মস্তিষ্কের কার্যকারিতা, এবং শরীরের রোগ প্রতিরোধ ক্ষমতা বাড়াতে গুরুত্বপূর্ণ ভূমিকা পালন করে থাকে। গ্রীষ্মমন্ডলীয় অঞ্চলে বসবাসরত শিশুদের মধ্যে পরিবেশজনিত আন্ত্রিক অকার্যকারিতা নামে একটি অন্ত্রের ব্যাধি দেখা যায়। সাম্প্রতিক কার্নিটিনের ঘাটতি এবং পরিবেশজনিত আন্ত্রিক অকার্যকারিতা একসাথে অল্প বয়স্ক শিশুদের বৃদ্ধি এবং বিকাশের উপর নেতিবাচক প্রভাব ফেলতে পারে। লেভোকার্নিটিন সিরাপ অপুষ্টিজনিত বাচ্চাদের জন্য একটি নতুন চিকিৎসা যুক্ত করতে পারে। ডাউন সিনড্রোম এবং হৃদরোগের চিকিৎসায় এই ওষুধ নিরাপদ বলে বিবেচিত হয়েছে।

৯ থেকে ২৪ মাস বয়সী পেডিয়াট্রিক রোগীর জনসংখ্যায় লেভোকার্নিটিন সিরাপের প্রতিক্রিয়া মূল্যায়ন করতে আমরা একটি গবেষণা পরিচালনা করছি এবং বিশ্ব স্বাস্থ্য সংস্থা (WHO) কর্তৃক অনুমোদিত অপুষ্টি চিকিৎসা সেবার প্রোটোকল এর সাথে অতিরিক্ত কার্যকারিতাটির তুলনা করছি। আমরা পরিবেশজনিত আন্ত্রিক অকার্যকারিতা এর চিকিৎসায় লেভোকার্নিটিন সিরাপের প্রভাবও মূল্যায়ন করবো।

**আপনাকে কেন এই গবেষণায় অংশগ্রহণ করার জন্য আহবান করছিঃ**

আপনার সন্তান যেহেতু তীব্র অপুষ্টিতে আক্রান্ত এবং ৯-২৪ মাস বয়সী তাই আমরা আমাদের গবেষণায়ে আপনার সন্তানের অংশগ্রহনের জন্য আপনাকে আমন্ত্রন জানাচ্ছি। আমরা আমাদের গবেষণার জন্য তার মত বাচ্চাদের সন্ধান করছি। যেহেতু আপনার বাচ্চার মারাত্মক তীব্র অপুষ্টি রয়েছে এবং পুষ্টি পুনর্বাসন ইউনিটে (NRU) বিশেষ সুবিধা ভিত্তিক ব্যবস্থাপনার প্রয়োজন রয়েছে. তাই আমরা ধরে নিই যে বিশ্ব স্বাস্থ্য সংস্থা (WHO) কর্তৃক অনুমোদিত অপুষ্টি চিকিৎসা সেবার সাথে সিরাপ লেভোকার্নিটিন যুক্ত করার মাধ্যমে ওজন বাড়ার হার বাড়বে এবং হাসপাতালের থাকার সময়কাল হ্রাস হওয়ার সাথে সাথে পরিবেশজনিত আন্ত্রিক অকার্যকারিতা এর অবস্থারও উন্নতি হবে। এই তথ্যগুলি যদি পাওয়া যায় তবে চিকিৎসকদের অপুষ্টি এবং পরিবেশজনিত আন্ত্রিক অকার্যকারিতা এর পরিচালনায় নতুন মাত্রা যুক্ত করতে সহায়তা করবে। এই কারণেই আমরা আপনাকে অনুরোধ করছি আপনার সন্তানকে আমাদের গবেষণায় অন্তর্ভুক্ত করার অনুমতি দিয়ে আমাদের সহায়তা করুন।

**পদ্ধতি এবং কার্যপ্রণালীঃ**

যদি আপনার সন্তনাকে আমাদের গবেষণায় অংশগ্রহনের অনুমতি দেন, তাহলে নিম্নলখিত কিছু করনীয় প্রত্যাশা করতে পারেনঃ

- এই হসাপাতালের ডাক্তার, নার্স এবং অন্যান্য কর্মীরা আপনার সন্তানকে সেবা ও সুচিকিৎসা প্রদান করবে। এই গবেষণায় অংশগ্রহণের জন্য কোন ভাবেই হাস্পাতালের মানসম্মত চিকিৎসার কোন পরিবর্তন হবে না এবং যদি ব্যবস্থাপনার জন্য প্রয়োজন হয় হাসপাতালের নীতি ও গাইডলাইন অনুযায়ী আমরা গবেষণাগারে পরীক্ষা নিরীক্ষা করাব।
- আমরা আপনাকে আপনার সন্তানের অসুস্থতা সম্পর্কিত কিছু প্রশ্ন করব এবং হাসপাতালে ভর্তির সময় ও ভর্তি থাকাকালীন প্রতিদিন তার অবস্থা (অগ্রগতি এবং অবনতি) পর্যবেক্ষণের জন্য শারীরিক পরীক্ষা করব।
- আমরা বিশ্ব স্বাস্থ্য সংস্থা (WHO) কর্তৃক অনুমোদিত অপুষ্টির চিকিৎসা সেবার পাশাপাশি লেভোকার্নিটিন সিরাপ বা প্লাসবো দিনে তিনবার দিব মোট ১৫ দিনের জন্য। এই ওষুধগুলো সম্পূর্ণ নিরাপদ।
- আমরা গবেষণায় অন্তর্ভুক্তির দিন এবং চিকিৎসা সমাপ্তির ১৫ তম দিনে শিশুর প্রস্রাব এবং মলের নমুনা সংগ্রহ করব এবং চিকিৎসা সমাপ্তির ১৮০ তম দিনে শুধুমাত্র শিশুর মলের নমুনা সংগ্রহ করব।
- আমরা গবেষণায় অন্তর্ভুক্তির দিন এবং চিকিৎসা সমাপ্তির ১৫ তম দিনে ৫ মিলি রক্তের নমুনাও সংগ্রহ করব। সুঁই এবং সিরিঞ্জের মাধ্যমে শিরা (cubital vein) থেকে রক্ত সংগ্রহ করা হবে।
- চিকিৎসা শেষ হওয়ার পর হাসপাতালের ডাক্তার আপনার সন্তানকে হাসপাতালের নিয়ম অনুযায়ী ছুটি দিবেন। এর মানে আপনার সন্তানের চিকিৎসা আমাদের গবেষণার কারণে প্রভাবিত হয়নি।
- গবেষণাকার্যে আপনার সহযোগীতা খুবই প্রয়োজন।

**ঝুঁকি এবং সুবিধাঃ**

**অপেক্ষিত সম্ভাব্য ঝুঁকিঃ**

এই গবেষণায় অংশগ্রহণে আপনার সন্তানের কোন মারাত্মক ঝুঁকি নেই। সম্ভাব্য প্রতিকূল ঘটনাগুলো হচ্ছে, বমি বমি ভাব, বমি, ডায়রিয়া। সর্তকতা অবলম্বনের পরেও যদি এই গবেষণার ফলে আপনার সন্তানের যে কোন উপসর্গ দেখা দেয় আমরা ঢাকা হাসপাতাল, আইসিডিডিআরবিতে (কলেরা হাসপাতালে) আপনার সন্তানের উপযুক্ত চিকিৎসা দিব। আপনার সন্তান রক্ত প্রদানের সময় সামান্য অস্বস্তি বা ব্যাথা অনুভব করতে পারে। রক্ত প্রদানের ক্ষেত্রে কোন মারাত্মক ঝুঁকি নেই।

**অপেক্ষিত সম্ভাব্য সুবিধাঃ**

আপনার শিশু প্রত্যক্ষ এবং পরোক্ষভাবে এই গবেষণায় অংশ নেওয়া থেকে উপকৃত হবে। আমরা ধারনা করছি পুষ্টি উপাদান এল-কার্নিটিন এর অভাবে আপনার বাচ্চা পুষ্টিহিনতায় এবং পরিবেশজনিত আন্ত্রিক অকার্যকারিতায় আক্রান্ত। এই গবেষণায় অংশগ্রহনের মাধ্যমে আপনি আপনার বাচ্চার এল-কার্নিটিন এবং পরিবেশজনিত আন্ত্রিক অকার্যকারিতার বর্তমান অবস্থা সম্পর্কে জানতে পারবেন। তদুপরি, আপনার শিশু অপুষ্টির জন্য আরও কার্যকর চিকিৎসা এবং পরিবেশজনিত আন্ত্রিক অকার্যকারিতা এর জন্য নতুন চিকিৎসার বিকাশ করতে আমাদের বোঝাপড়ায় অবদান রাখতে সক্ষম হবে। দীর্ঘমেয়াদে, এই অধ্যয়নের ফলাফলগুলি অপুষ্টি ও পরিবেশজনিত আন্ত্রিক অকার্যকারিতা আক্রান্ত শিশুদের উপর লেভোকার্নিটিন সিরাপ পরিপূরকতার প্রভাবগুলি বুঝতে আমাদের বাংলাদেশকে এবং অন্যান্য দেশ কেও উপকার করবে।

**গোপনীয়তা ও বিশ্বস্ততাঃ**

আমরা অত্যন্ত দৃঢ়তার সাথে জানাচ্ছি যে, আপনার দেয়া সকল তথ্য অত্যন্ত গোপনীয়তার সাথে সংরক্ষণ করা হবে এতে আপনার সন্তানের নাম বা পরিচয় প্রকাশ পাবে না। আমরা আপনার সন্তানের স্বাস্থ্য, চিকিৎসা বিবরণ, এবং গবেষণাগারে পরীক্ষার সকল তথ্য অত্যন্ত গোপনীয়তার সহিত রাখব। এই গবেষণার গবেষকবৃন্দ, নিয়ন্ত্রক কর্তৃপক্ষ ও নৈতিক পর্যালোচনাকারী কমিটি (একদল অভিক্ত ব্যক্তিবর্গ যারা গবেষণায় অংশগ্রহণকারীদের স্বার্থ রক্ষা করেন), গবেষণার দাতা ব্যক্তিরা এই তথ্য দেখতে। এই গবেষণার ফলাফল বিশ্লেষণ অথবা প্রকাশের সময় বাচ্চার নাম ও পরিচয় প্রকাশ করা হবে না।

**ভবিষ্যতে তথ্যের ব্যবহারঃ**

ভবিষ্যতে এই গবেষণায় পাওয়া তথ্য গোপনীয়তা সহকারে ব্যবহার হবে। আমরা আপনার সন্তানের প্রস্রাবের, মলের এবং রক্তের নমুনাও গোপনীয়তার সাথে সংরক্ষণ করবো। আমরা নমুনাগুলোর আরো উচ্চতর পরীক্ষার মাধ্যমে ওষুধের প্রভাব সম্পর্কে জানতে পারব। এধরনের গবেষণার জন্য পুণরায় কোন সম্মতির জন্য অনুরোধ করা হবে না। কোন ব্যবসায়িক উদ্দেশ্যে এই গবেষণার তথ্য ব্যবহৃত হবে না।

**গবেষনায় অংশগ্রহন না করার অধিকারঃ**

এই গবেষণায় আপনার সন্তানের অংশগ্রহণ ঐচ্ছিক ব্যাপার। আপনার সম্পূর্ণভাবে অধিকার আছে এই গবেষণায় আপনার সন্তানের অংশগ্রহন করানো বা না করানো। আপনি চাইলে আপনার সন্তানকে গবেষণা চলাকালীন যে কোন সময়ে কোন কারন না দেখিয়ে গবেষণা হতে সরিয়ে নিতে পারেন। গবেষণায় অন্তর্ভুক্তির পর আপনি যদি গবেষণা থেকে সরে যাওয়ার সিদ্ধান্ত গ্রহণ করেন সে ক্ষেত্রে ইতিমধ্যে যে সকল জৈবিক নমুনা/তথ্য সংগ্রহ করা হয়েছিল তা আমরা সংরক্ষণ করব এবং ভবিষ্যৎ গবেষণার কাজে ব্যবহার করব, সে ক্ষেত্রেও আপনার নাম বা পরিচয় প্রকাশ করা হবে না। আপনি গবেষণায় অংশগ্রহণ করতে না চাইলে অথবা গবেষণা হতে সন্তানকে সরিয়ে নিলেও কোন শাস্তি হবে না অথবা সেবা, সুবিধা বা মনোযোগ হতে বঞ্চিত হবেন না।

**ক্ষতিপূরণ/প্রদেয়ঃ**

এই হাসপাতালে সকল রোগীর বিনামূল্যে চিকিৎসা দেয়া হয় এবং আপনার সন্তানের ক্ষেত্রেও এর ব্যতিক্রম হবে না। একইভাবে আমাদের গবেষণায় আপনার সন্তানের অংশগ্রহণের জন্য কোন অর্থ আপনাকে প্রদান করা হবে না। যদি আপনার সন্তানের গবেষণা সংক্রান্ত কোন ক্ষতি হয় তবে ঢাকা হাসপাতাল (কলেরা হাসপাতাল), মহাখালীতে মানসম্মত চিকিৎসা পাবে। গবেষণায় অংশগ্রহনের সাপেক্ষে হাসপাতালে ১৫ তম দিনে এবং চিকিৎসা সমাপ্তির ১৮০ তম দিনে আসার প্রয়োজনে যাতায়াত খরচ প্রদান করা হবে।

**সম্মতি পত্র (Main Trial)**

| Protocol No: 21046 | Version No: 1.00 | Date: 24.05.2021 |
| --- | --- | --- |

**গবেষণা প্রকল্পের শিরোনামঃ** Role of L-Carnitine supplementation on rate of weight gain and biomarkers of Environmental Enteric Dysfunction (EED) in children with severe acute malnutrition

**প্রধান গবেষকের নামঃ**- ডাঃ জিনাত আলম

**প্রতিষ্ঠানঃ** - আইসিডিডিআর,বি

আপনি আপনার সন্তানকে যদি এই গবেষণায় অংশগ্রহন করাতে আগ্রহী হন তাহলে নিম্নের নির্ধারিত স্থানে টিক (√)) চিহ্ন দিন এবং সবশষে নির্ধারিত স্থানে স্বাক্ষর অথবা বাম বৃদ্ধাঙ্গুলীর ছাপ দিন।

- আমি অংশগ্রহনকারীর অবহিতকরন পত্রের ভার্সন 1.০০ পড়েছি,

আমার এই গবেষণা সম্পর্কে প্রশ্ন করার এবং আলোচনা করার সুযোগ ছিল এবং

আমি আমার প্রশ্নের সন্তোষজনক উত্তর পেয়েছি। হ্যাঁ না

- আমি বুঝেছি যে, কোন কারন ছাড়াই আমার সন্তানকে নিয়ে গবেষণা থেকে বিরত থাকার স্বাধীনতা

আমার আছে। হ্যাঁ না

- আমি আমার সন্তানের কাছ থেকে ০৫ মি. লি রক্ত দিতে সম্মত আছি। হ্যাঁ না
- আমি আমার সন্তানের কাছ থেকে মলের নমুনা দিতে সম্মত আছি। হ্যাঁ না
- আমি আমার সন্তানের কাছ থেকে মুত্রের নমুনা দিতে সম্মত আছি। হ্যাঁ না
- আমি আমার সন্তানের রক্তের, মলের, এবং মুত্রের নমুনা সমুহ পরবর্তী গবেশনার জন্য সংরক্ষণে সম্মত আছি। হ্যাঁ না
- আমি বুঝেছি যে, আমার দেয়া তথ্যাবলী গোপনীয়। হ্যাঁ না
- আমার সন্তানের সনাক্তকরন যোগ্য তথ্যসমূহ ভবিষ্যতে নৈতিকভাবে অনুমোদিত গবেষণা সমূহে

ব্যবহারে আমি সম্মত আছি। হ্যাঁ না

- এই গবেষণার সাথে সম্পর্কিত ভবিষ্যত গবেষণার জন্য আমার সাথে যোগাযোগের ব্যপারে

আমি সম্মত আছি। হ্যাঁ না

- আমি এও বুঝেছি যে, এই গবেষণা চলাকালীন সংগৃহীত আমার সন্তানের মেডিক্যাল নোট এবং তথ্যাদির

প্রাসঙ্গিক অংশ প্রয়োজনে স্পন্সর বা নিয়ন্ত্রনকারী কর্তৃপক্ষ থেকে ক্ষমতাপ্রাপ্ত ব্যক্তিরা দেখতে

পারেন, যা এই গবেষণায় আমার সন্তানের অংশগ্রহনের সাথে সম্পর্কিত। ঐ সকল ব্যক্তিদের আমার

সন্তানের তথ্যসমূহ দেখার অনুমতি প্রদান করছি। হ্যাঁ না

- আমি এই গবেষণায় আমার সন্তানকে অংশগ্রহন করাতে সম্মত আছি। হ্যাঁ না

| ...........................................  অংশগ্রহনকারীর স্বাক্ষর/বাম বৃদ্ধাঙ্গুলির ছাপ | ...........................................  অংশগ্রহনকারীর নাম | ..........................  তারিখ |
| --- | --- | --- |
| ...........................................  সাক্ষীর স্বাক্ষর/বাম বৃদ্ধাঙ্গুলির ছাপ | ...........................................  সাক্ষীর নাম | ..........................  তারিখ |
| ...........................................  গবেষক/তথ্য সংগ্রহকারীর স্বাক্ষর | ...........................................  গবেষক/তথ্য সংগ্রহকারীর নাম | ..........................  তারিখ |

**যোগাযোগঃ**

আপনার কোনো প্রশ্ন থাকলে আপনি এখনই আমাকে জিজ্ঞাসা করতে পারেন। এছাড়াও যদি গবেষণা সম্পর্কিত আরো কোন প্রশ্ন, আপত্তি বা অভিযোগ থাকে তাহলে নীচের ঠিকানায় সরাসরি যোগাযোগ করতে পারেন।

| যোগাযোগের উদ্দেশ্য | নাম ও পরিচয় | যোগাযোগের ঠিকানা |
| --- | --- | --- |
| গবেষণা সম্পর্কিত কোন প্রশ্ন, অনুসন্ধান বা অসুবিধা | ডাঃ জিনাত আলম  প্রকল্প গবেষণা চিকিৎসক ও প্রধান গবেষক | কলেরা হাসপাতাল, মহাখালি, ঢাকা-১২১২  মোবাইলঃ ০১৬৭৬৭৪২২৫৫ |
| গবেষণা সম্পর্কিত কোন অসন্তোষ বা অভিযোগ থাকলে | এম এ সালাম খান (আই আর বি কো-অরডিনেটর সেক্রেটারিয়েট) | কলেরা হাসপাতাল, মহাখালি, ঢাকা-১২১২  মোবাইল: (সকাল ৯.০০- বিকাল ৫.০০): ৯৮২৭০৮৪ অথবা ০১৭১১৪২৮৯৮৯ |

আপনার সহযোগিতার জন্য ধন্যবাদ।

**Appendix 4**

| Protocol No: 21046 | Version No: 1.00 | Date: 24.05.2021 |
| --- | --- | --- |

**WMA DECLARATION OF HELSINKI – ETHICAL PRINCIPLES FOR MEDICAL RESEARCH INVOLVING HUMAN SUBJECTS**

Adopted by the 18th WMA General Assembly, Helsinki, Finland, June 1964
and amended by the:
29th WMA General Assembly, Tokyo, Japan, October 1975
35th WMA General Assembly, Venice, Italy, October 1983
41st WMA General Assembly, Hong Kong, September 1989
48th WMA General Assembly, Somerset West, Republic of South Africa, October 1996
52nd WMA General Assembly, Edinburgh, Scotland, October 2000
53rd WMA General Assembly, Washington DC, USA, October 2002 (Note of Clarification added)
55th WMA General Assembly, Tokyo, Japan, October 2004 (Note of Clarification added)
59th WMA General Assembly, Seoul, Republic of Korea, October 2008
64th WMA General Assembly, Fortaleza, Brazil, October 2013

**Preamble**

1. The World Medical Association (WMA) has developed the Declaration of Helsinki as a statement of ethical principles for medical research involving human subjects, including research on identifiable human material and data.

The Declaration is intended to be read as a whole and each of its constituent paragraphs should be applied with consideration of all other relevant paragraphs.

2. Consistent with the mandate of the WMA, the Declaration is addressed primarily to physicians. The WMA encourages others who are involved in medical research involving human subjects to adopt these principles.

**General Principles**

3. The Declaration of Geneva of the WMA binds the physician with the words, “The health of my patient will be my first consideration,” and the International Code of Medical Ethics declares that, “A physician shall act in the patient’s best interest when providing medical care.”

4. It is the duty of the physician to promote and safeguard the health, well-being and rights of patients, including those who are involved in medical research. The physician’s knowledge and conscience are dedicated to the fulfillment of this duty.

5. Medical progress is based on research that ultimately must include studies involving human subjects.

6. The primary purpose of medical research involving human subjects is to understand the causes, development and effects of diseases and improve preventive, diagnostic and therapeutic interventions (methods, procedures and treatments). Even the best proven interventions must be evaluated continually through research for their safety, effectiveness, efficiency, accessibility and quality.

7. Medical research is subject to ethical standards that promote and ensure respect for all human subjects and protect their health and rights.

8. While the primary purpose of medical research is to generate new knowledge, this goal can never take precedence over the rights and interests of individual research subjects.

9. It is the duty of physicians who are involved in medical research to protect the life, health, dignity, integrity, right to self-determination, privacy, and confidentiality of personal information of research subjects. The responsibility for the protection of research subjects must always rest with the physician or other health care professionals and never with the research subjects, even though they have given consent.

10. Physicians must consider the ethical, legal and regulatory norms and standards for research involving human subjects in their own countries as well as applicable international norms and standards. No national or international ethical, legal or regulatory requirement should reduce or eliminate any of the protections for research subjects set forth in this Declaration.

11. Medical research should be conducted in a manner that minimizes possible harm to the environment.

12. Medical research involving human subjects must be conducted only by individuals with the appropriate ethics and scientific education, training and qualifications. Research on patients or healthy volunteers requires the supervision of a competent and appropriately qualified physician or other health care professional.

13. Groups that are underrepresented in medical research should be provided appropriate access to participation in research.

14. Physicians who combine medical research with medical care should involve their patients in research only to the extent that this is justified by its potential preventive, diagnostic or therapeutic value and if the physician has good reason to believe that participation in the research study will not adversely affect the health of the patients who serve as research subjects.

15. Appropriate compensation and treatment for subjects who are harmed as a result of participating in research must be ensured.

**Risks, Burdens and Benefits**

16. In medical practice and in medical research, most interventions involve risks and burdens.

Medical research involving human subjects may only be conducted if the importance of the objective outweighs the risks and burdens to the research subjects.

17. All medical research involving human subjects must be preceded by careful assessment of predictable risks and burdens to the individuals and groups involved in the research in comparison with foreseeable benefits to them and to other individuals or groups affected by the condition under investigation.

Measures to minimize the risks must be implemented. The risks must be continuously monitored, assessed and documented by the researcher.

18. Physicians may not be involved in a research study involving human subjects unless they are confident that the risks have been adequately assessed and can be satisfactorily managed.

When the risks are found to outweigh the potential benefits or when there is conclusive proof of definitive outcomes, physicians must assess whether to continue, modify or immediately stop the study.

**Vulnerable Groups and Individuals**

19. Some groups and individuals are particularly vulnerable and may have an increased likelihood of being wronged or of incurring additional harm.

All vulnerable groups and individuals should receive specifically considered protection.

20. Medical research with a vulnerable group is only justified if the research is responsive to the health needs or priorities of this group and the research cannot be carried out in a non-vulnerable group. In addition, this group should stand to benefit from the knowledge, practices or interventions that result from the research.

**Scientific Requirements and Research Protocols**

21. Medical research involving human subjects must conform to generally accepted scientific principles, be based on a thorough knowledge of the scientific literature, other relevant sources of information, and adequate laboratory and, as appropriate, animal experimentation. The welfare of animals used for research must be respected.

22. The design and performance of each research study involving human subjects must be clearly described and justified in a research protocol.

The protocol should contain a statement of the ethical considerations involved and should indicate how the principles in this Declaration have been addressed. The protocol should include information regarding funding, sponsors, institutional affiliations, potential conflicts of interest, incentives for subjects and information regarding provisions for treating and/or compensating subjects who are harmed as a consequence of participation in the research study.

In clinical trials, the protocol must also describe appropriate arrangements for post-trial provisions.

**Research Ethics Committees**

23. The research protocol must be submitted for consideration, comment, guidance and approval to the concerned research ethics committee before the study begins. This committee must be transparent in its functioning, must be independent of the researcher, the sponsor and any other undue influence and must be duly qualified. It must take into consideration the laws and regulations of the country or countries in which the research is to be performed as well as applicable international norms and standards but these must not be allowed to reduce or eliminate any of the protections for research subjects set forth in this Declaration.

The committee must have the right to monitor ongoing studies. The researcher must provide monitoring information to the committee, especially information about any serious adverse events. No amendment to the protocol may be made without consideration and approval by the committee. After the end of the study, the researchers must submit a final report to the committee containing a summary of the study’s findings and conclusions.

**Privacy and Confidentiality**

24. Every precaution must be taken to protect the privacy of research subjects and the confidentiality of their personal information.

**Informed Consent**

25. Participation by individuals capable of giving informed consent as subjects in medical research must be voluntary. Although it may be appropriate to consult family members or community leaders, no individual capable of giving informed consent may be enrolled in a research study unless he or she freely agrees.

26. In medical research involving human subjects capable of giving informed consent, each potential subject must be adequately informed of the aims, methods, sources of funding, any possible conflicts of interest, institutional affiliations of the researcher, the anticipated benefits and potential risks of the study and the discomfort it may entail, post-study provisions and any other relevant aspects of the study. The potential subject must be informed of the right to refuse to participate in the study or to withdraw consent to participate at any time without reprisal. Special attention should be given to the specific information needs of individual potential subjects as well as to the methods used to deliver the information.

After ensuring that the potential subject has understood the information, the physician or another appropriately qualified individual must then seek the potential subject’s freely-given informed consent, preferably in writing. If the consent cannot be expressed in writing, the non-written consent must be formally documented and witnessed.

All medical research subjects should be given the option of being informed about the general outcome and results of the study.

27. When seeking informed consent for participation in a research study the physician must be particularly cautious if the potential subject is in a dependent relationship with the physician or may consent under duress. In such situations the informed consent must be sought by an appropriately qualified individual who is completely independent of this relationship.

28. For a potential research subject who is incapable of giving informed consent, the physician must seek informed consent from the legally authorized representative. These individuals must not be included in a research study that has no likelihood of benefit for them unless it is intended to promote the health of the group represented by the potential subject, the research cannot instead be performed with persons capable of providing informed consent, and the research entails only minimal risk and minimal burden.

29. When a potential research subject who is deemed incapable of giving informed consent is able to give assent to decisions about participation in research, the physician must seek that assent in addition to the consent of the legally authorized representative. The potential subject’s dissent should be respected.

30. Research involving subjects who are physically or mentally incapable of giving consent, for example, unconscious patients, may be done only if the physical or mental condition that prevents giving informed consent is a necessary characteristic of the research group. In such circumstances the physician must seek informed consent from the legally authorized representative. If no such representative is available and if the research cannot be delayed, the study may proceed without informed consent provided that the specific reasons for involving subjects with a condition that renders them unable to give informed consent have been stated in the research protocol and the study has been approved by a research ethics committee. Consent to remain in the research must be obtained as soon as possible from the subject or a legally authorized representative.

31. The physician must fully inform the patient which aspects of their care are related to the research. The refusal of a patient to participate in a study or the patient’s decision to withdraw from the study must never adversely affect the patient-physician relationship.

32. For medical research using identifiable human material or data, such as research on material or data contained in biobanks or similar repositories, physicians must seek informed consent for its collection, storage and/or reuse. There may be exceptional situations where consent would be impossible or impracticable to obtain for such research. In such situations the research may be done only after consideration and approval of a research ethics committee.

**Use of Placebo**

33. The benefits, risks, burdens and effectiveness of a new intervention must be tested against those of the best proven intervention(s), except in the following circumstances:

Where no proven intervention exists, the use of placebo, or no intervention, is acceptable; or

Where for compelling and scientifically sound methodological reasons the use of any intervention less effective than the best proven one, the use of placebo, or no intervention is necessary to determine the efficacy or safety of an intervention

And the patients who receive any intervention less effective than the best proven one, placebo or no intervention will not be subject to additional risks of serious or irreversible harm as a result of not receiving the best proven intervention.

Extreme care must be taken to avoid abuse of this option.

**Post-Trial Provisions**

34. In advance of a clinical trial, sponsors, researchers and host country governments should make provisions for post-trial access for all participants who still need an intervention identified as beneficial in the trial. This information must also be disclosed to participants during the informed consent process.

**Research Registration and Publication and Dissemination of Results**

35. Every research study involving human subjects must be registered in a publicly accessible database before recruitment of the first subject.

36. Researchers, authors, sponsors, editors and publishers all have ethical obligations with regard to the publication and dissemination of the results of research. Researchers have a duty to make publicly available the results of their research on human subjects and are accountable for the completeness and accuracy of their reports. All parties should adhere to accepted guidelines for ethical reporting. Negative and inconclusive as well as positive results must be published or otherwise made publicly available. Sources of funding, institutional affiliations and conflicts of interest must be declared in the publication. Reports of research not in accordance with the principles of this Declaration should not be accepted for publication.

**Unproven Interventions in Clinical Practice**

37.       In the treatment of an individual patient, where proven interventions do not exist or other known interventions have been ineffective, the physician, after seeking expert advice, with informed consent from the patient or a legally authorized representative, may use an unproven intervention if in the physician’s judgment it offers hope of saving life, re-establishing health or alleviating suffering. This intervention should subsequently be made the object of research, designed to evaluate its safety and efficacy. In all cases, new information must be recorded and, where appropriate, made publicly available.

# Check-List

**Check-list for Submission of Research Protocol**

**For Consideration of the Research Review Committee (RRC)**

**[Please check all appropriate boxes]**

| 1. Has the proposal been reviewed, discussed and cleared by all listed investigators? |
| --- |
| Yes  No |
| If the response is No, please clarify the reasons: |
| 1. Has the proposal been peer-reviewed externally? |
| Yes  No  External Review Exempted |
| If the response is ‘No’ or “External Review Exempted”, please explain the reasons: |
| If the response is “Yes”, please indicate if all of their comments have been addressed? |
| Yes (please attach)  No (please indicate reason(s)): |
| 1. Has the budget been reviewed and approved by icddr,b’s Finance?   Yes  No (reason): ________________________________________________________________ |
| 1. Has the Ethics Certificate(s) been attached with the Protocol?   Yes  No  If the answer is ‘No’, please explain the reasons: |
| 24.05.2021 Signature of the Principal Investigator Date |
