# Supplementary material for: Role of L-Carnitine supplementation on rate of weight gain and biomarkers of Environmental Enteric Dysfunction in children with severe acute malnutrition: A protocol for a double-blinded randomized controlled trial
Source: PLoS One. 2022 Sep 30;17(9):e0275291. doi: 10.1371/journal.pone.0275291 (PMC9524645; doi:10.1371/journal.pone.0275291)
Supplement: Attachment — Submitted filename: comments.doc [file pone.0275291.s003.doc]

**Review comments on Manuscript Number: PONE-D-22-11684. Entitled " Role of L-Carnitine supplementation on rate of weight gain and biomarkers of Environmental Enteric Dysfunction in children with severe acute malnutrition: a protocol for a double-blinded randomized controlled trial"**

Overall, the idea of research is very interesting, well written and reasonable. However, there are some comments and suggestions.

**Title :** Well structured

**Abstract:**

- Background is recommended to be more concise.
- Keywords in alphabetical order.

**Introduction:** Well structured

**Methodology** **:** Well structured

**Statistical analysis:** Well structured

**Discussion**: Well structured
