# Supplementary material for: Role of L-Carnitine supplementation on rate of weight gain and biomarkers of Environmental Enteric Dysfunction in children with severe acute malnutrition: A protocol for a double-blinded randomized controlled trial
Source: PLoS One. 2022 Sep 30;17(9):e0275291. doi: 10.1371/journal.pone.0275291 (PMC9524645; doi:10.1371/journal.pone.0275291)
Supplement: Attachment — Submitted filename: Response to Reviewers.docx [file pone.0275291.s004.docx]

Response to the Academic Editor's comments:

1. Please ensure that your manuscript meets PLOS ONE's style requirements, including those for file naming.**Response: Dear editor, thank you very much for your comment. File naming was edited to comply with the style requirements. We hopefully have no divergences from the style requirements now.**

2. Your ethics statement should only appear in the Methods section of your manuscript. If your ethics statement is written in any section besides the Methods, please move it to the Methods section and delete it from any other section. Please ensure that your ethics statement is included in your manuscript, as the ethics statement entered into the online submission form will not be published alongside your manuscript. **Response: Dear editor, thank you very much for your suggestion. We moved our ethics statement into the method section after deleting it from the other area (Page:5; Line:100-105).**

3. Please include captions for your Supporting Information files at the end of your manuscript, and update any in-text citations to match accordingly. **Response: Dear editor, thank you for your suggestion. We included captions for our supporting information files at the end of our revised manuscript (Page:18, Line:377-381), and the in-text citation was matched accordingly** **(Page:5,16; Line:103,105,317).**

4. We note that the original protocol file you uploaded contains a confidentiality notice indicating that the protocol may not be shared publicly or be published. Please note, however, that the PLOS Editorial Policy requires that the original protocol be published alongside your manuscript in the event of acceptance. Please note that should your paper be accepted, all content including the protocol will be published under the Creative Commons Attribution (CC BY) 4.0 license, which means that it will be freely available online, and any third party is permitted to access, download, copy, distribute, and use these materials in any way, even commercially, with proper attribution.

Therefore, we ask that you please seek permission from the study sponsor or body imposing the restriction on sharing this document to publish this protocol under CC BY 4.0 if your work is accepted. We kindly ask that you upload a formal statement signed by an institutional representative clarifying whether you will be able to comply with this policy. Additionally, please upload a clean copy of the protocol with the confidentiality notice (and any copyrighted institutional logos or signatures) removed. **Response: Dear editor, thank you for your advice. We changed the confidentiality notice and agreed to share our protocol publicly.**

5. Please review your reference list to ensure that it is complete and correct. If you have cited papers that have been retracted, please include the rationale for doing so in the manuscript text, or remove these references and replace them with relevant current references. Any changes to the reference list should be mentioned in the rebuttal letter that accompanies your revised manuscript. If you need to cite a retracted article, indicate the article's retracted status in the References list and also include a citation and full reference for the retraction notice. **Response: Dear editor, thank you for your advice. We reviewed our reference list. We hope it is completed and correct now (Page:17-18; Line:333-375).**

Response to the Reviewer's comments:

Comments to the Author
1. Does the manuscript provide a valid rationale for the proposed study, with clearly identified and justified research questions?

The research question outlined is expected to address a valid academic problem or topic and contribute to the base of knowledge in the field.

Reviewer #1: Yes

Reviewer #2: Yes

**Response: Dear reviewers, thank you very much for your positive comment.**

2. Is the protocol technically sound and planned in a manner that will lead to a meaningful outcome and allow testing the stated hypotheses?

The manuscript should describe the methods in sufficient detail to prevent undisclosed flexibility in the experimental procedure or analysis pipeline, including sufficient outcome-neutral conditions (e.g. necessary controls, absence of floor or ceiling effects) to test the proposed hypotheses and a statistical power analysis where applicable. As there may be aspects of the methodology and analysis which can only be refined once the work is undertaken, authors should outline potential assumptions and explicitly describe what aspects of the proposed analyses, if any, are exploratory.

Reviewer #1: Yes

Reviewer #2: Yes

**Response: Thank you for your comment.**

3. Is the methodology feasible and described in sufficient detail to allow the work to be replicable?

Descriptions of methods and materials in the protocol should be reported in sufficient detail for another researcher to reproduce all experiments and analyses. The protocol should describe the appropriate controls, sample size calculations, and replication needed to ensure that the data are robust and reproducible.

Reviewer #1: Yes

Reviewer #2: Yes

**Response: Thank you so much for your valuable opinion.**

4. Have the authors described where all data underlying the findings will be made available when the study is complete?

The [PLOS Data policy](https://journals.plos.org/plosone/s/materials-and-software-sharing) requires authors to make all data underlying the findings described in their manuscript fully available without restriction, with rare exception, at the time of publication. The data should be provided as part of the manuscript or its supporting information, or deposited to a public repository. For example, in addition to summary statistics, the data points behind means, medians and variance measures should be available. If there are restrictions on publicly sharing data—e.g. participant privacy or use of data from a third party—those must be specified.

Reviewer #1: Yes

Reviewer #2: Yes

**Response: Thank you so much for your comment.**

5. Is the manuscript presented in an intelligible fashion and written in standard English?

PLOS ONE does not copyedit accepted manuscripts, so the language in submitted articles must be clear, correct, and unambiguous. Any typographical or grammatical errors should be corrected at revision, so please note any specific errors here.

Reviewer #1: Yes

Reviewer #2: Yes

**Response: Thank you very much for your opinion.**

6. Review Comments to the Author

Reviewer #1: Dear Author,
Thank you for your very interesting effort. Your finding is very important to the community and I found the detail of your work and it is coined in very excellent manner. Please keep up this fruitful work. Please also check grammatical error in some few place of the work around result and discussion.

**Response to Reviewer #1: Thank you for your valuable suggestion and inspiration. We have checked and sorted the grammatical error in the revised manuscript as you suggested (Page:14-15, Line:283-303).**

Reviewer #2: Review comments on Manuscript Number: PONE-D-22-11684. Entitled " Role of L-Carnitine supplementation on rate of weight gain and biomarkers of Environmental Enteric Dysfunction in children with severe acute malnutrition: a protocol for a double-blinded randomized controlled trial"
Overall, the idea of research is very interesting, well written and reasonable. However, there are some comments and suggestions.
Title: Well structured
Abstract:
- Background is recommended to be more concise.
- Keywords in alphabetical order.
Introduction: Well structured
Methodology: Well structured
Statistical analysis: Well structured
Discussion: Well structured

**Response to Reviewer #2: Thank you for your valuable suggestions. We concised the background of the abstract and reordered keywords alphabetically in the revised manuscript (Page:2-3; Line:26-34, 48-49).**
